# In Situ Construction of Interface with Photothermal and Mutual Catalytic Effect for Efficient Solar‐Driven Reversible Hydrogen Storage of MgH_2_

**DOI:** 10.1002/advs.202400274

**Published:** 2024-03-22

**Authors:** Xuechun Hu, Xiaowei Chen, Xiaoyue Zhang, Yang Meng, Guanglin Xia, Xuebin Yu, Dalin Sun, Fang Fang

**Affiliations:** ^1^ Department of Materials Science Fudan University Shanghai 200433 P. R. China; ^2^ Department of Physics Jimei University Xiamen 361021 P. R. China; ^3^ Yiwu Research Institute of Fudan University Yiwu Zhejiang 322000 P. R. China

**Keywords:** hydrogen storage, magnesium hydride, mutual catalytic effect, photothermal effect, solar energy

## Abstract

Hydrogen storage in MgH_2_ is an ideal solution for realizing the safe storage of hydrogen. High operating temperature, however, is required for hydrogen storage of MgH_2_ induced by high thermodynamic stability and kinetic barrier. Herein, flower‐like microspheres uniformly constructed by N‐doped TiO_2_ nanosheets coated with TiN nanoparticles are fabricated to integrate the light absorber and thermo‐chemical catalysts at a nanometer scale for driving hydrogen storage of MgH_2_ using solar energy. N‐doped TiO_2_ is in situ transformed into TiN_x_O_y_ and Ti/TiH_2_ uniformly distributed inside of TiN matrix during cycling, in which TiN and Ti/TiH_x_ pairs serve as light absorbers that exhibit strong localized surface plasmon resonance effect with full‐spectrum light absorbance capability. On the other hand, it is theoretically and experimentally demonstrated that the intimate interface between TiH_2_ and MgH_2_ can not only thermodynamically and kinetically promote H_2_ desorption from MgH_2_ but also simultaneously weaken Ti─H bonds and hence in turn improve H_2_ desorption from the combination of weakened Ti─H and Ti─H bonds. The uniform integration of photothermal and catalytic effect leads to the direct action of localized heat generated from TiN on initiating the catalytic effect in realizing hydrogen storage of MgH_2_ with a capacity of 6.1 wt.% under 27 sun.

## Graphical abstract

This work provides a rational design to realize the reversible hydrogen storage of MgH_2_ using sustainable solar energy based on the uniform integration of unexpected photothermal and catalytic effect that leads to the direct action of localized heat generated from TiN on initiating the mutual catalytic effect of TiN_x_O_y_ and Ti/TiH_2_ pairs with MgH_2_.

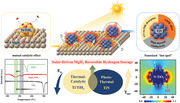

## 1 Introduction

Hydrogen as a versatile energy carrier is considered as a key component for developing the decarbonization strategy of energy storage, power generation, and chemical industry.^[^ ^1^ ^]^ The safe and efficient storage and transportation of hydrogen is a vital bottleneck for the large‐scale application of hydrogen energy.^[^ ^2^ ^]^ In this regard, MgH_2_, a typical solid‐state light‐weight metal hydride, has attracted tremendous attention due to its high theoretical gravimetric and volumetric hydrogen storage capacity (i.e., 7.6 wt.% and 110 g H_2_ L^−1^, respectively), low cost, and environmental friendliness.^[^ ^3^ ^]^ However, a theoretical operating temperature of over 280 °C is required for reversible hydrogen storage of MgH_2_ owing to its high thermodynamic stability, and the practical application is even worse induced by the high kinetic barriers that further increase the operating temperature up to over 350 °C.^[^ ^4^ ^]^ It not only limits the practical application of MgH_2_ due to the mismatch of the operating conditions required for reversible hydrogen storage and most current fuel cells but also results in the consumption of a large amount of extra energy for thermal heating.^[^ ^5^ ^]^ Moreover, the traditional energy supply relies mainly on external electric heating equipment, which further reduces the practical hydrogen storage density and hence energy utilization efficiency of the whole system.^[^ ^6^ ^]^

Solar energy, which is clean, sustainable sufficient to fulfill the energy demand, is usually considered as a promising alternative to fossil fuels.^[^ ^7^ ^]^ Solar‐driven reversible hydrogen storage of metal hydrides could effectively regulate the activity of H_2_ adsorption and desorption reaction, which opens up a new avenue for the wide use of renewable solar power to realize the reversible storage of H_2_.^[^ ^8^ ^]^ It is realized based on the combination of the photothermal effect of a light absorber that generates localized heat for increasing the temperature of MgH_2_ and catalytic effect of thermo‐chemical catalysts that is able to decrease the operating temperature required for the reversible hydrogen storage of MgH_2_. The localized heat generated by the photothermal effect should be able to match the operating temperature required for driving reversible hydrogen storage of MgH_2_ under the catalysis of thermo‐chemical catalysts that are physically separated from light absorbers. The physical phase separation between the light absorber and catalytic sites not only postpones the action of heat generated by the photothermal effect on initiating the catalytic effect in improving the hydrogen storage performance of MgH_2_ but also simultaneously results in the loss of heat during transfer process, which leads to poor catalytic effect and low light‐to‐heat efficiency. As a result, an ultrahigh light intensity of 4.0 W cm^−2^ is still required to realize a reversible hydrogen storage reaction of MgH_2_.^[^ ^9^ ^]^

Inspired by the above progress, it is expected that the homogeneous integration of advanced photothermal agents and catalytic sites is a theoretically ideal design to realize solar‐driven reversible hydrogen storage. Herein, flower‐like microspheres comprising uniformly constructed N‐doped TiO_2_ nanosheets coated with TiN nanoparticles (referred to as TiN@N‐TiO_2_) are fabricated to integrate light absorption and thermochemical catalysis at a nanometer scale. During the initial mixing process with MgH_2_, the N‐doped TiO_2_ undergoes an in situ transformation into TiN_x_O_y_ and TiH_2_ nanoparticles, which are uniformly distributed inside the TiN matrix. Both TiN and TiH_2_ serve as light absorbers, exhibiting a strong localized surface plasmon resonance (LSPR) effect with full‐spectrum light absorption capability. This results in a temperature of 283 °C under a light intensity of only 19 sun, which is over 112 °C higher than that achieved with TiO_2_ alone. On the other hand, it is theoretically and experimentally demonstrated that the introduction of TiH_2_ clusters thermodynamically promotes the detachment of H atoms from MgH_2_ by breaking Mg─H bonds and forming new Ti─H bonds within TiH_2_ matrix. Additionally, the spontaneous transfer of H atom from MgH_2_ into TiH_2_ simultaneously weakens Ti─H bonds and hence kinetically facilitates H_2_ desorption from the combination of weakened Ti─H and Ti─H bonds. As a result, the homogeneous distribution of TiN_x_O_y_ and TiH_2_ nanoparticles within the TiN matrix facilitates the integration of photothermal and catalytic effects on a nanometer scale, which enables the direct utilization of localized heat to initiate the catalytic activity of TiN_x_O_y_ and TiH_2_ nanoparticles, thereby realizing solar‐driven hydrogen storage of MgH_2_ with a reversible capacity of 6.1 wt.% under a light intensity of 27 sun.

## 2 Results and Discussion

**Figure 1:**
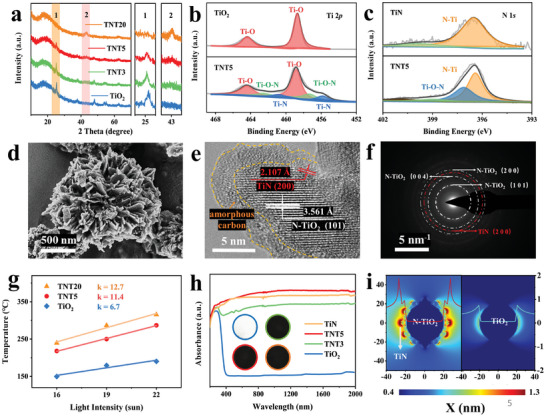
a) XRD patterns of the as‐synthesized of TiO_2_ and TiN@N‐TiO_2_. b) High‐resolution Ti 2*p* XPS spectrum of TiO_2_ and TNT5. c) High‐resolution N 1*s* XPS spectrum of TNT5 and TiN. d) SEM, e) HRTEM images, and f) SAED pattern of TNT5. g) The relationship between the temperature and the light intensity for TiO_2_, TNT5, and TiN after an irradiation time of 15 min. h) UV–vis–NIR absorption spectra of TiO_2_, TNT3, TNT5, and TiN (inset: digital photos of blue – TiO_2_, green – TNT3, red – TNT5, and orange – TiN). i) Theoretical FDTD simulated localized electric field enhancement profiles of TiN@N‐TiO_2_, with TiO_2_ included for comparison.

As schematically illustrated in Figure S1 (Supporting Information), the flower‐like TiO_2_ microspheres were first synthesized by the solvothermal reaction of tetrabutyl titanate in acetic acid and the subsequent calcination at 500 °C.^[^ ^10^ ^]^ Subsequently, flower‐like N‐doped TiO_2_ microspheres uniformly coated with TiN nanoparticles could be obtained after the calcination of mixed TiO_2_ and melamine under an H_2_/N_2_ atmosphere. By adjusting the mass ratio between TiO_2_ and melamine (MA) (1:*x*, *x* = 3, 5, 20, respectively), denoted as TNT*x*, the amount of thus‐formed TiN could be facile tuned. The coexistence of N‐doped anatase TiO_2_ (N‐TiO_2_) and TiN was validated by X‐ray diffraction (XRD) patterns (**Figure** **1**a) in the as‐synthesized TNT*x*. As the mass ratio of melamine increases, the diffraction peaks of anatase TiO_2_ at 25°, 47°, 53°, and 55° gradually weaken in intensity and exhibit a slight shift toward lower 2*θ* degrees. These observations suggest the replacement of O^2−^ ions by larger N^3−^ ions and/or the insertion of nitrogen atoms into the TiO_2_ lattice, resulting in the formation of N‐doped TiO_2_.In addition, new diffraction peaks appearing at 42° and 63°, attributed to the (200) and (220) plane reflections of TiN, become evident as the reaction between TiO_2_ and melamine progresses. Moreover, the X‐ray photoelectron spectroscopy (XPS) survey illustrates the presence of Ti 2*p*, O 1*s*, and C 1*s* peaks in the as‐synthesized TiO_2_ (Figure S2a, Supporting Information) and the characteristic peaks of N 1*s* at 396–400 eV in the as‐synthesized TNT5, confirming the successful doping of N atoms into TiO_2_. In addition to the characteristic peaks of TiO_2_ at 458.7/464.5 eV, the presence of peaks corresponding to the Ti─O─N bond at 457.3/462.6 eV and the Ti─N bond of TiN at 455.8/460.7 eV^[^ ^11^ ^]^ could be observed in high‐resolution Ti 2*p* XPS spectrum of TNT5, which provides additional evidence for the coexistence of N‐TiO_2_ and TiN in TNT5. This result coincides well with the high‐resolution N 1*s* and O 1*s* XPS results,^[^ ^12^ ^]^ which also validates the presence of interstitial N atoms and O vacancies for adjusting unbalanced electron distribution due to the introduction of N^3−^ (Figure 1c; Figure S2b, Supporting Information).

Scanning electron microscopy (SEM) image (Figure S3, Supporting Information) verifies that the as‐synthesized TiO_2_ exhibits flower‐like hierarchical microspheres with a diameter of ≈1–1.5 µm assembled by densely stacked uniform nanosheets with a thickness of 20 nm. High‐resolution TEM (HRTEM) image of the as‐synthesized TiO_2_ illustrates the presence of the (101) plane of TiO_2_ with a typical *d*‐spacing of 3.53 Å (Figure S4, Supporting Information), corresponding well with XRD results. After the nitridation process, the flower‐like hierarchical microspheres of TiO_2_ are well preserved (Figure 1d). The observation of the lattice spacing of 3.56 Å corresponding to the (101) plane of N‐doped TiO_2_ at the center of the nanoparticles and the lattice spacing of 2.10 Å corresponding to the (200) plane of TiN on the surface of nanoparticles in the HRTEM image (Figure 1e) provides direct evidence to the formation of TiN uniformly decorated on the surface of TiO_2_, which corresponds well with the selected area electron diffraction (SAED) results (Figure 1f). As the nitridation process progresses, there is a noticeable emergence of TiN nanoparticles forming and adhering to the surface of TiO_2_ nanosheets. These nanoparticles subsequently aggregate, resulting in the development of wrinkled flower‐like morphologies, as depicted in Figure S3 (Supporting Information). The energy dispersive X‐ray spectroscopy (EDS) elemental mapping (Figure S5, Supporting Information) confirms the homogeneous distribution of Ti, N, O, and C elements, which further confirms the uniform coverage of TiN on the surface of N‐doped TiO_2_ in TNT*x*. Moreover, an amorphous carbon layer with a thickness of ≈2 nm is observed on the surface of TiN@N‐TiO_2_ due to the retention of certain carbon atoms from melamine under inert conditions (Figure 1e). Considering EDS and TG results (Figures S6 and S7, Supporting Information), the weight content of N in TNT5 is determined to be 5.4 wt.%, and the ratio of N is tunable by adjusting the proportion of melamine (Table S1, Supporting Information). Moreover, an amorphous carbon layer with a thickness of ≈2 nm is observed on the surface of TiN@N‐TiO_2_ due to the retention of certain carbon atoms from melamine under inert conditions (Figure 1e). The progressive generation of TiN particles and the deposition of an amorphous carbon layer contribute to a gradual elevation in the specific surface area of TiO_2_ from 72.8 to 107.1 m^2^ g^−1^. Simultaneously, there is a slight reduction in the average pore size from 23.4 to 19.1 nm attributed to the occupation of pore space by in situ formation of TiN nanoparticles (Figure S8, Supporting Information).

After the nitridation process, an obvious change of color could be visually observed (the inset of Figure 1h). The initially synthesized TiO_2_ exhibits a pristine white color, whereas following nitridation, its coloration deepens to a profound black, resembling the appearance of commercial TiN, indicating the ability to absorb sunlight across the entire spectrum as evidenced by the UV–vis–NIR absorbance spectrum (Figure 1h). Typically, a weak broad absorption peak between 400 and 600 nm could be observed for TiN due to its localized surface plasmon resonance (LSPR) effect^[^ ^13^ ^]^ and the absorption curve of TiO_2_ indicates strong UV light harvesting capability and weak vis–NIR light absorbance with an absorption edge at only 400 nm. By comparison, a better UV‐light absorbance and a redshift of the absorption edge are observed from TNT3. Based on the plots of the Kubelka–Munk remission function^[^ ^14^ ^]^ according to the adsorption curves of TiO_2_ and TNT3, the bandgap of the as‐synthesized TiO_2_ is calculated to be 2.97 eV, while this value is significantly reduced to 1.89 eV for TNT3 (Figure S9, Supporting Information). This result directly demonstrates the enhancement of the light absorption capability of TiO_2_ via the doping of N that leads to the formation of O vacancies, which could effectively narrow the bandgap of TiO_2_ by introducing additional energy levels within the band structure.^[^ ^15^ ^]^ With the increased formation of excellent light‐absorbing TiN, the light absorption of TNT5 in the UV–vis range has further improved (Figure 1h).

To clarify the mechanism of plasmon‐enhanced photothermal properties induced by TiN, the cross‐sectional electric field distributions of TNT*x* and pure TiO_2_ are theoretically investigated using the finite‐difference time‐domain (FDTD) simulation (Figure 1i). The profiles of electric fields are obtained when the 525 nm incident light is perpendicular to one of the facets. Localized “hot spots”^[^ ^16^ ^]^ could be observed clearly around TiN nanoparticles and in the interfacial regions between TiN and TiO_2_. The presence of TiN notably extends the reach of the surrounding electric field, thereby confirming the strong LSPR effect of TiN in enhancing the photothermal performance of TNT*x*. Consistent with the full‐spectrum absorbance trend, the LSPR effect of TNT*x* could also enlarge the area of localized “hot spots” around TiN nanoparticles, even in the near‐infrared range, as demonstrated by the adoption of 1200 nm incident light (Figure S10, Supporting Information), which offers further evidence of the broadened light absorption capability of TiO_2_ through the nitridation process. As a result, under a light irradiation intensity of 19 sun, the surface temperature of TiO_2_ reaches 171 °C only within 15 min due to its limited light absorption ability. By comparison, a high temperature of 241 and 283 °C could be obtained for TNT5 and TNT20, respectively, which directly demonstrates the superior photothermal properties induced by the nitridation process. In addition, a linear relationship between the surface temperature and light intensity in the range of 16–22 sun could be observed and as expected, TiO_2_ after nitridation exhibits higher photothermal conversion efficiency than that of pure TiO_2_ (Figure 1g). Maintaining an unaltered composition and microstructure after light irradiation, it is of great importance that nitrided TiO_2_ demonstrates stable and efficient light absorption and photothermal conversion capabilities (Figure S11, Supporting Information).

**Figure 2:**
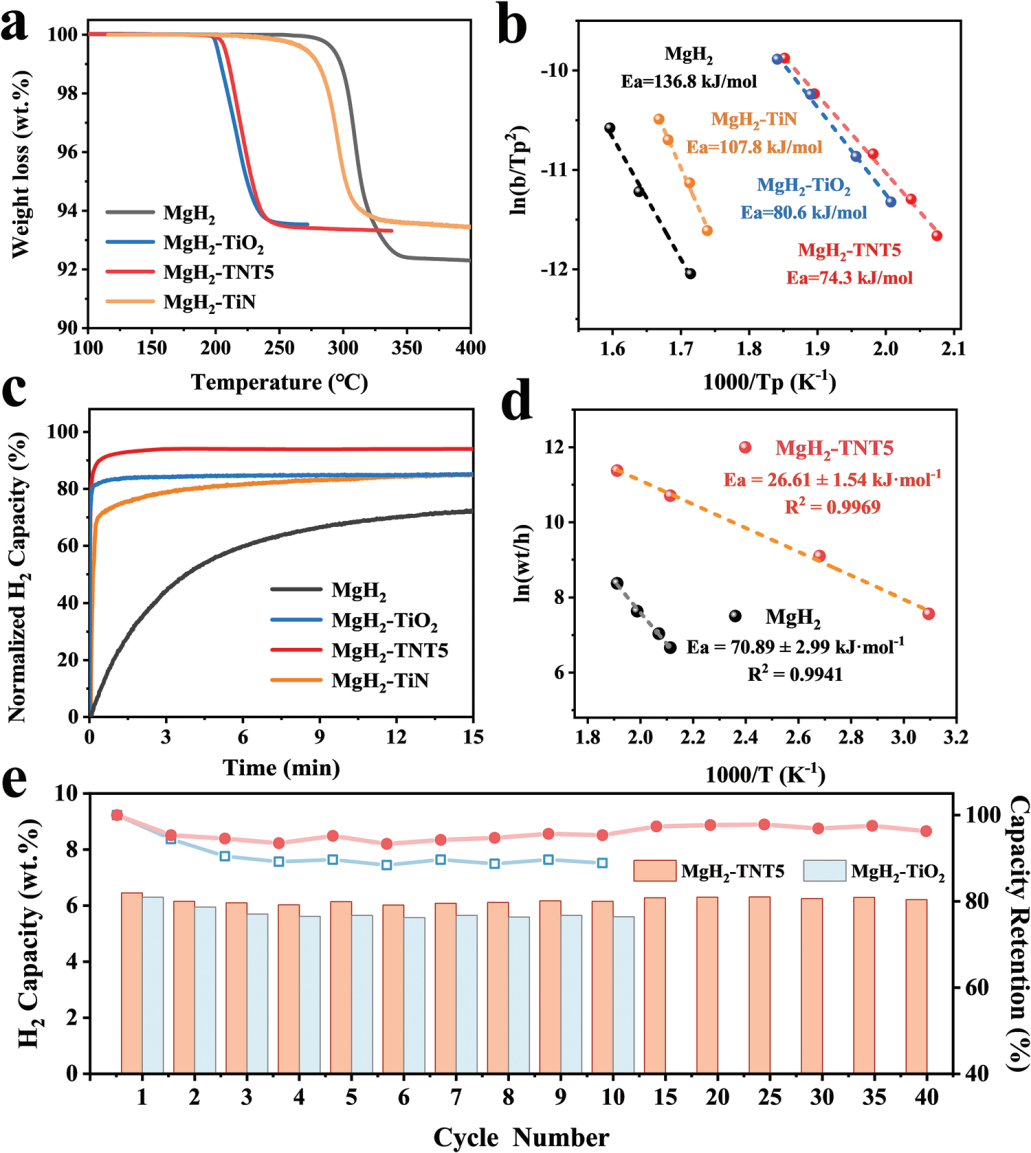
a) H_2_ desorption curves and b) Kissinger's plots of MgH_2_ under the catalysis of TiO_2_, TNT5, and TiN, respectively, with ball‐milled MgH_2_ included for comparison. c) Isothermal H_2_ adsorption curves of MgH_2_ at 250 °C under the catalysis of TNT5 and TiO_2_, respectively, with ball‐milled MgH_2_ included for comparison. d) The activation energy for hydrogenation of MgH_2_ catalyzed by TNT5 is fitted by the JMA equation. e) Cycling isothermal H_2_ desorption curves and capacity retention of MgH_2_ under the catalysis of TNT5 and TiO_2_ at 260 °C.

The hydrogen storage performance of MgH_2_ under the presence of TiO_2_ before and after nitridation with a weight ratio of 10 wt.% is first investigated using traditional external thermal heating via temperature‐programmed desorption (TPD) method (**Figure** **2**a). Owing to the sluggish kinetics and high thermal stability of reversible hydrogen storage of MgH_2_, a high operating temperature of 300 °C is required for the dehydrogenation of MgH_2_.Under the catalysis of TiO_2_, MgH_2_ is capable of releasing H_2_ at 198 °C with a peak temperature of 215 °C, indicating the effective catalytic role of TiO_2_ in enhancing the hydrogen storage performance of MgH_2_. By comparison, the peak temperature for the dehydrogenation of MgH_2_ catalyzed by TiN reaches 290 °C, which is comparable to that of the ball‐milled MgH_2_, indicating the limited catalytic effect of TiN in improving H_2_ desorption of MgH_2_. Under the catalysis of TiO_2_ after nitridation, the peak temperature for the dehydrogenation of MgH_2_ could be maintained at ≈220 °C, with a terminal temperature of 250 °C, which directly demonstrates the superior catalytic effect of TNT*x* in promoting H_2_ desorption of MgH_2_. Among them, the apparent activation energy (*E*_a_) of MgH_2_ catalyzed TNT5 calculated by the Kissinger's equation that is determined to be 74.3 kJ mol^−1^ is the lowest, which is 60 kJ mol^−1^ lower than that of ball‐milled MgH_2_ (Figure 2b; Figure S12, Table S2, Supporting Information). It provides direct evidence of the effective role of TNT5 in catalytically improving H_2_ desorption of MgH_2_.

Upon the reversible hydrogenation process, the effective role of TNT5 in optimizing H_2_ adsorption of Mg could also be observed. Specifically, 5.92 wt.% H_2_ could be charged within 30 min under 5 MPa H_2_ at 250 °C, corresponding to 90.7% of its maximum capacity, while this value is decreased to 80.8% and 76.9% for MgH_2_ catalyzed by TiO_2_ and ball‐milled MgH_2_ under identical conditions (Figure 2c). Upon decreasing the temperature down to 100 and 50 °C, 5.03 and 3.54 wt.% of H_2_ could still be recharged into dehydrogenated MgH_2_ catalyzed by TNT5 within 30 min (Figure S13, Supporting Information). According to the Johnson–Mehl–Avrami (JMA) equation,^[^ ^17^ ^]^ the *E*_a_ for the hydrogenation of MgH_2_ catalyzed by TNT5 is calculated to be 26.6 kJ mol^−1^, much lower than that of bulk MgH_2_ (Figure 2d). Hence, considering the superior photothermal performance and catalytic effect, TNT5 is selected for driving reversible hydrogen storage of MgH_2_ using solar energy.

Impressively, a reversible capacity of 6.21 wt.% H_2_, corresponding to a capacity retention of ≈95.8%, could be retained for MgH_2_ catalyzed by TNT5 after 40 cycles of H_2_ desorption and adsorption, while only a capacity retention of 88.9% could be achieved for MgH_2_ catalyzed by TiO_2_ after 10 cycles (Figure 2e). This result validates the excellent catalytic stability of TNT5 in improving reversible hydrogen storage of MgH_2_ as supported by the repeated transformation between Mg and MgH_2_ in XRD results (Figure S14, Supporting Information).

**Figure 3:**
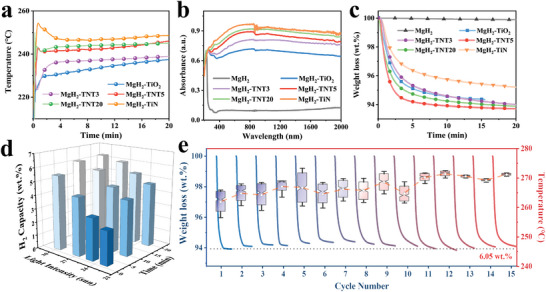
a) The surface temperatures of MgH_2_ under the catalysis of TNT5, TiO_2_, and TiN, respectively, with a light intensity of 27 sun. b) UV–vis–NIR absorption spectra of MgH_2_ under the catalysis of TiO_2_, TNT5, and TiN, respectively, with ball‐milled MgH_2_ included for comparison. c) Solar‐driven H_2_ desorption of MgH_2_ under the catalysis of TiO_2_, TNT5, and TiN, including ball‐milled MgH_2_ for comparison. d) H_2_ desorption curves of MgH_2_ under the catalysis of TNT5 under various light intensities over time. e) Cycling H_2_ desorption curves of MgH_2_ catalyzed by TNT5 under a light intensity of 35 sun and the corresponding surface temperature after 5–10 min irradiation.

Based on the remarkable photothermal capability of TNT5 and the exceptional thermal‐driven hydrogen storage performance of MgH_2_ under the catalysis of TNT5, it is expected that the thermal energy generated by TNT5 upon light irradiation is capable of driving reversible hydrogen storage of MgH_2_. Specifically, under a light intensity of 27 sun, the surface temperature of MgH_2_ catalyzed by TNT5 could reach 240 °C within 2 min, which is much higher than that of TiO_2_ (i.e., 225 °C) and, however, lower than that of TiN (i.e., 250 °C). This result is consistent with the observation from the UV–vis–NIR absorption test (**Figure** **3**a,b), in which the average absorbance of MgH_2_ was gradually increased with the addition of TiO_2_ after nitridation, particularly as the relative TiN content rises, from 0.678 of MgH_2_ catalyzed by TiO_2_ to 0.769 of TNT3, 0.822 of TNT5, and 0.864 of TNT20, and finally, MgH_2_ catalyzed by TiN shows the highest light absorbance capability in the full spectral range, especially in the region of vis–NIR, with an average absorbance of up to 0.897.

In response to the irradiation with a light intensity of 27 sun, only neglectable H_2_ could be released from the ball‐milled MgH_2_ within 60 min owing to its limited photothermal capability (Figure 3c), while a capacity of 4.5 wt.% could be released from MgH_2_ after the addition of TiN with excellent photothermal performance that leads to a high surface temperature of 250 °C. This result validates the effective role of the photothermal effect in realizing solar‐driven hydrogen storage of MgH_2_. On the other hand, although the photothermal capability of TiO_2_ is much lower than that of TiN, MgH_2_ catalyzed by TiO_2_ is able to release 5.7 wt.% of hydrogen, even 1.2 wt.% higher than that under the catalysis of TiN, which validates the key role of catalytic effect in realizing hydrogen storage of MgH_2_ using solar energy. Therefore, it is essential to carefully adjust the content of TiO_2_ and TiN to optimize the solar‐driven reversible hydrogen storage of MgH_2_. Induced by the comparable photothermal performance of TNT5 with TiN and the superior catalytic effect of TNT5 than TiO_2_, MgH_2_ under the catalysis of TNT5 is capable of releasing 6.1 wt.% H_2_ within 30 min under a light intensity of 27 sun. As expected, the surface temperatures of MgH_2_ catalyzed by TNT5 exhibit a linear dependence on light intensity. Specifically, the surface temperatures of 220, 230, 240, and 250 °C are achieved as the light intensity increases to 23, 25, 27, and 30 sun, respectively, which consistently remain 5–10 °C higher than those observed for TiO_2_ under identical conditions (Figure S15a, Supporting Information), This provides direct evidence of the effective role of N‐doping in enhancing the photothermal effect of TiO_2_, thereby promoting its catalytic activity in achieving solar‐driven hydrogen storage performance of MgH_2_. In particular, 5.5 wt.% H_2_ could be released within 2 min from MgH_2_ catalyzed by TNT5, and a complete dehydrogenation with a capacity of 6.2 wt.% could be achieved within 12 min under a light intensity of 30 sun (Figure 3d), showing significantly faster and more sufficient dehydrogenation performance than that of TiO_2_ (Figure S15b–d, Supporting Information), which is the best reported photothermal catalyst for solar‐driven MgH_2_ hydrogen storage materials so far (Table S3, Supporting Information).

Solar‐driven reversible hydrogen storage of MgH_2_, which is a critically challenging issue for its practical application, is subsequently investigated under a light intensity of 35 sun (Figure 3e). A reversible capacity of 6.05 wt.% could still be achieved for MgH_2_ catalyzed by TNT5 after 15 cycles, corresponding to a capacity retention of 95%. It is worth noting that the H_2_ desorption of MgH_2_ catalyzed by TNT5 is accelerated after 15 cycles with a slight increase in surface temperature (Figure S16, Supporting Information). The light absorbance in the vis and NIR regions of MgH_2_ under the catalysis of TNT5 and TiO_2_ is enhanced after several cycles, while the absorbance in the UV range is decreased, especially for MgH_2_ catalyzed by TiO_2_ (Figure S17a, Supporting Information). High‐resolution XPS results demonstrate that, after mixing with MgH_2_ and the repeated H_2_ desorption and adsorption process, TiO_2_ and N‐doped TiO_2_ are reduced to Ti/TiH_x_ (Figures S18–S20, Supporting Information), which, in comparison with TiO_2_, exhibit superior absorbance in the vis–NIR region and inferior light absorbance in the UV region.^[^ ^18^ ^]^ It is consistent with the weakening of the light absorbance of MgH_2_ catalyzed by TiO_2_ and TNT5 in the UV region after cycling (Figure S17a,b, Supporting Information). Induced by superior light absorption performance, the surface temperature of MgH_2_ after the addition of pure Ti and TiH_2_ is higher than that of TiO_2_, which could further promote its catalytic effect in improving hydrogen storage of MgH_2_ (Figure S17c, Supporting Information). However, it could be expected that nano‐scaled Ti/TiH_2_ pairs generated by in situ reduction reaction between MgH_2_ and TiO_2_ would construct closer contact interfaces with MgH_2_ than by physical introduction, leading to better H_2_ desorption performance, thus highlighting the additional advantages of in situ nano‐sized catalysts in improving solar‐driven hydrogen storage of MgH_2_ (Figure S17d, Supporting Information). In addition, as the cycling process progresses, a decreasing fluctuation range of surface temperature within 5–10 min in Figure 3e indicates that in situ Ti/TiH_2_ assists MgH_2_ in facilitating quicker attainment of stable temperature and achieving faster dehydrogenation by enhancing the photothermal conversion efficiency.

In order to unveil the catalytic effect of TNT5 in enhancing the hydrogen storage performance of MgH_2_, XPS measurement is conducted to investigate the phase change during repeated H_2_ desorption and adsorption processes. The characteristic XPS peaks of metallic Ti at 454 eV could be observed with the absence of TiO_2_ after the ball milling process between MgH_2_ and TNT5 (Figure S18, Supporting Information), indicating the formation of metallic Ti from the reduction of N‐doped TiO_2_ by MgH_2_ during the ball milling process. After the reversible hydrogenation process, characteristic peaks belonging to Ti─H bonds at 453 and 459.8 eV could be found with the absence of Ti, confirming the reversible formation of Ti/TiH_2_ species during the cycling process of MgH_2_. High‐resolution Ti 2*p* and N 1*s* XPS results of MgH_2_ catalyzed by TNT5 at different states (Figure S19, Supporting Information) demonstrate that the characteristic peaks of TiN and TiN_x_O_y_^[^ ^19^ ^]^ inside of TNT5 could be well‐preserved throughout the reversible hydrogen storage process. This result demonstrates the in situ reversible formation of Ti/TiH_2_ and the well‐preservation of TiN and TiN_x_O_y_ upon repeated H_2_ desorption and adsorption process that could play a catalytic effect in improving the hydrogen storage performance of MgH_2_. XPS results of MgH_2_ cycling under light irradiation with the addition of TNT5 also come to the same conclusion (Figure S20, Supporting Information), which strengthens the credibility of the above conclusion. Considering that Ti/TiH_2_ could also be observed during the cycling process of MgH_2_ catalyzed by TiO_2_ that exhibits a comparable catalytic effect with TNT5 with the observation of only a limited catalytic effect for TiN (Figure S21, Supporting Information), it is concluded that Ti/TiH_2_, as well as TiN_x_O_y_, plays an important catalytic role in improving hydrogen storage performance of MgH_2_.

**Figure 4:**
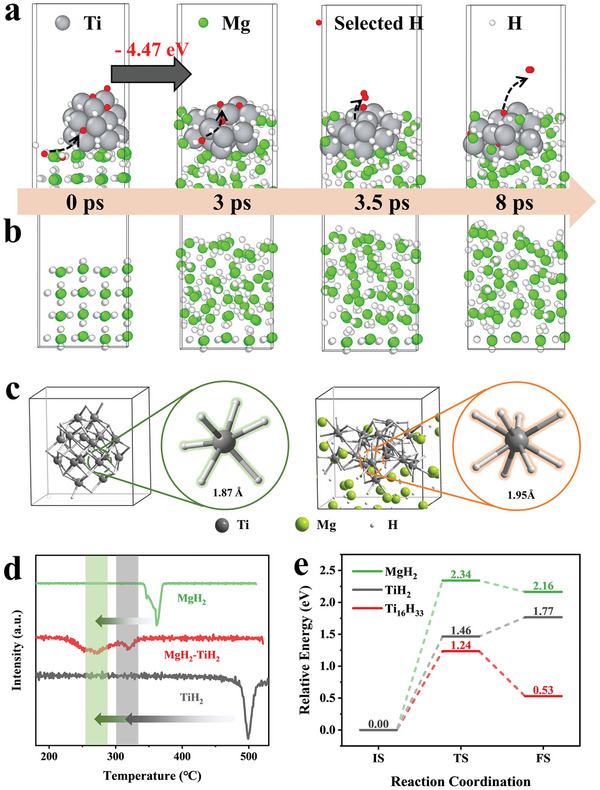
a,b) Snapshot of the AIMD simulation of the H_2_ desorption from MgH_2_ under the catalysis of TiH_2_ and pristine MgH_2_, respectively. c) The bond length of Ti─H bond in TiH_2_ clusters without and with the presence of MgH_2_. d) H_2_ desorption derivative curves of ball‐milled MgH_2_, TiH_2_, and MgH_2_ with the addition of TiH_2_. e) Energy profile of the self‐dehydrogenation of MgH_2_, TiH_2_, and Ti_16_H_33_.

Considering that the H_2_ desorption from MgH_2_ is an endothermic process with a large enthalpy of 74.1 kJ mol^−1^ while the reversible H_2_ adsorption of Mg is thermodynamically favorable and only TiH_2_ could be detected along the formation of MgH_2_, ab initio molecular dynamics (AIMD) simulations are conducted to unravel the effect of TiH_2_ in improving H_2_ desorption of MgH_2_ (**Figure** **4**a,b). Upon the proceeding of the simulation, only the formation of disordered structure could be observed for pristine MgH_2_ with no sign of H_2_ desorption. In strong contrast, after the introduction of TiH_2_ clusters with a particle size of ≈1 nm, numerous H atoms of MgH_2_ will be spontaneously not only detached from MgH_2_ matrix but also transferred into TiH_2_ toward the formation of new Ti─H bonds. First‐principles calculations based on density functional theory reveal that the hydrogen transference through Mg─H bond breaking and Ti─H new bond formation, along with the interface disorder reconstruction, is thermodynamically favorable with an enthalpy of −4.47 eV, which validates the effective role of TiH_2_ in thermodynamically promoting the dissociation and transfer diffusion of H atoms in MgH_2_ matrix. In addition, the energy barrier for H_2_ desorption from TiH_2_ is 1.46 eV, which is much lower than that of MgH_2_ (2.34 eV) (Figure 4e; Figure S22, Supporting Information). This suggests that dehydrogenation through the combination of Ti─H bonds is more favorable than the breaking of Mg─H bonds, indicating that the spontaneous transfer of H atoms from MgH_2_ to TiH_2_ could also kinetically promote H_2_ desorption from MgH_2_. As a result, H_2_ desorption would be observed on the surface of TiH_2_ clusters after only 3.5 ps from the start of the simulation.

The average length of Ti─H bonds in pristine TiH_2_ clusters is 1.87 Å, while this value is increased to 1.95 Å in the presence of MgH_2_, indicating that the spontaneous transfer of H atom from MgH_2_ to TiH_2_ could in turn weaken Ti─H bonds (Figure 4c). Hence, after the introduction of one H atom into TiH_2_, a lower energy barrier of only 1.24 eV for the H_2_ desorption from the H‐enriched TiH_2_ nanoclusters could be observed (Figure 4e). It could be further supported by the experimental results (Figure 4d), in which the peak temperature for H_2_ desorption from both MgH_2_ and TiH_2_ in the mixed composite of MgH_2_ and TiH_2_ is much lower than their bulk counterpart. Specifically, MgH_2_ could achieve complete dehydrogenation before 300 °C, and even TiH_2_, whose initial dehydrogenation temperature is originally as high as 470 °C, could achieve partial dehydrogenation to form TiH_1.5_, as depicted in Figure S23 (Supporting Information). This result demonstrates the intimate interface between TiH_2_ and MgH_2_ could not only thermodynamically and kinetically promote H_2_ desorption from MgH_2_ but also simultaneously weaken Ti─H bonds and hence in turn improve the H_2_ desorption from the combination of weakened Ti─H and Ti─H bonds, which coincides well with the observation of significant decrease of H_2_ desorption from MgH_2_ under the catalysis of TNT5 and TiO_2_. As a result, when the simulation time is increased from 3 to 8 ps, facile H_2_ desorbed from the surface of TiH_2_ with the spontaneous breaking of Mg─H bonds could be further observed. In addition to TiH_2_, the energy barrier for H_2_ desorption from MgH_2_ under the catalysis of TiN_x_O_y_ could also be lowered down to 0.61 eV (Figure S24, Supporting Information), 1.7 eV lower than that of pure MgH_2_, which validates the additional catalytic role of TiN_x_O_y_ in improving the H_2_ desorption of MgH_2_.

Elemental mapping results verify the uniform distribution of Ti and N elements inside of MgH_2_ matrix, indicating the homogeneous mixing of catalytic species with MgH_2_ during the cycling process (Figure S25, Supporting Information). HRTEM images reveal that uniform interfaces between TiH_2_ and TiN_x_O_y_ as the effective catalysts and TiN as the light absorber are well preserved, coupled with the homogeneous distribution of Ti/TiH_2_ and TiN*_x_* O_y_ inside of MgH_2_ matrix, which could promote the direct action of localized heat generated by TiN on activating the catalytic effect in improving H_2_ adsorption and desorption of MgH_2_.

**Figure 5:**
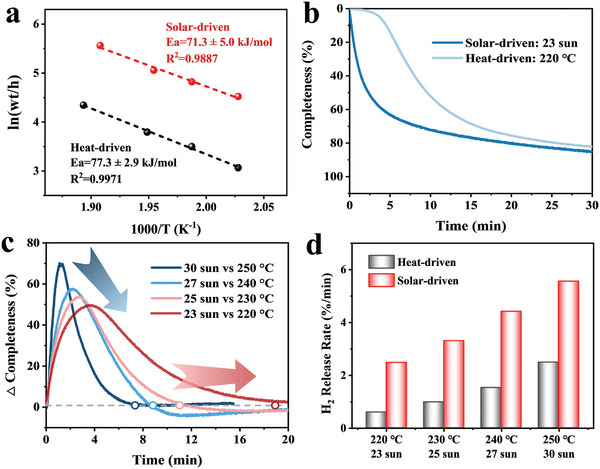
The comparison of a) apparent dehydrogenation activation energies and b) H_2_ desorption curves of MgH_2_ catalyzed by TNT5 under solar and electric heating. c) The difference in dehydrogenation completeness ratio over time in MgH_2_ catalyzed by TNT5 driven by light and at various light intensities and the corresponding temperatures. d) The comparison of the H_2_ release rate of MgH_2_ catalyzed by TNT5 using solar energy and thermal heating.

Subsequently, the comparison between light irradiation and traditional electric heating in driving hydrogen storage of MgH_2_ under the catalysis of TNT5 is investigated in detail, where the solar‐driven H_2_ desorption process using specific light intensity could be considered as an isothermal heating process. The activation energy of MgH_2_ catalyzed by TNT5 under electric heating calculated by Arrhenius equation according to isothermal hydrogen desorption profiles (**Figure** **5**a; Figure S26a, Supporting Information) is 77.3 ± 2.9 kJ mol^−1^, comparable to the value of 71.3 ± 5 kJ mol^−1^ calculated under the light irradiation. Therefore, it could be concluded that solar‐driven hydrogen storage in MgH_2_ is essentially a thermo‐catalytic process, where light only serves as a source of energy through photothermal conversion, without the additional role of photocatalysis. Despite this, compared to traditional electric heating, solar‐driven heating still exhibited remarkable hydrogen storage performance. Under the light intensity of 23 sun that corresponds to a surface temperature of 220 °C, MgH_2_ under the catalysis of TNT5 is capable of releasing 4.45 wt.% H_2_ (corresponding to 73% of its theoretical capacity) within 10 min, while more than 20 min is required by means of electrothermal heating as the energy input to reach the same level of H_2_ desorption (Figure 5b). This difference could be attributed to the significant heat loss that occurs during the conduction of heat from the heating device to MgH_2_ in electric heating, as well as the influence of MgH_2_ with poor thermal conductivity. As a result, the temperature rise within the MgH_2_ is slower. On the other hand, light can directly act on the uniformly dispersed photothermal agent TiN, enabling it to generate heat directly within the MgH_2_ matrix upon light irradiation, thus avoiding significant energy loss. Consequently, solar‐driven heating achieves not only a superior fast response to energy input within a few seconds but also a maximized energy utilization efficiency.

The difference between the desorption completeness ratio profiles over time driven by light and heating further highlights the advantages of solar‐driven over traditional heat‐driven hydrogen storage strategy (Figure S26b–d, Supporting Information). When 90% of H_2_ desorption is achieved under the light intensity of 30 sun, only 20% of H_2_ desorption could be obtained at 250 °C, in which the time to reach a maximum difference of 70% is only 2 min. However, when the light intensity is decreased to 23 sun, it takes 5 min to reach the maximum difference of 56%, not only the time is prolonged, but also the difference value is degraded. This result indicates that the stronger the light intensity, the more noticeable advantage in response rate and dehydrogenation rate of solar‐driven hydrogen storage over traditional heat‐driven hydrogen storage could be achieved. The solar‐driven method utilizes the localized heat generated by the LSPR effect of TiN that results in predictable higher temperature than the measured surface temperature of MgH_2_. Specifically under the light intensity of 23 sun, the measured temperature of MgH_2_ catalyzed by TNT5 is ≈220 °C, while TiN nanoparticles could even reach an instantaneous high temperature of 412 °C. This indicates that the localized temperature of the TiN in the composite is ≈180–230 °C higher than the overall temperature, demonstrating the strong photothermal conversion performance of the nanoscale photothermal agent (Figure S27, Supporting Information). Additionally, the close contact interface between the photothermic agent and the thermocatalysts allows for the direct action of heat on the catalytic species, further enhancing their catalytic activity on MgH_2_, which leads to superior peak dehydrogenation rate under light irradiation than that of the electric heating (Figure 5d). On the other hand, MgH_2_ with low thermal conductivity could act as a “heat isolator”,^[^ ^20^ ^]^ which not only reduces the temperature gradient near TiN nanoparticles but also minimizes the heat dissipation to the environment. This could further improve the light‐to‐heat efficiency of the photothermal effect in promoting overall solar‐driven hydrogen storage of MgH_2_.

However, solar‐driven hydrogen storage still faces the problem of poor thermal conductivity of hydrogen storage materials. Since the bottom of the pellet that cannot be directly irradiated by light needs to achieve dehydrogenation through heat conduction, poor thermal conductivity will lead to a serious tailing phenomenon of the dehydrogenation curve. Designing catalyst structures to improve the thermal conductivity is a typical strategy, but the ball milling process disrupts the catalyst's microstructure, which limits further exploration of its significance in the field of solar‐driven hydrogen storage from the perspective of structure design, and more attention is focused on the specific chemical composition's effects on photothermal and catalytic properties. In the future, nano‐confinement of hydrogen storage materials can be achieved on well‐designed structural carriers, such as flakes and cavities, which are recognized for their significant advantages in the photothermal and catalytic fields. Further exploration will shed light on the significance of catalyst structure in solar‐driven hydrogen storage.

## 3 Conclusion

In summary, flower‐liked microspheres constructed by N‐doped TiO_2_ uniformed coated with TiN nanoparticles are designed to integrate light absorbers and thermo‐chemical catalysts at a nanometer scale for realizing solar‐driven hydrogen storage of MgH_2_. After mixing with MgH_2_, N‐doped TiO_2_ are in situ transformed into TiN_x_O_y_, and Ti/TiH_2_ pairs are uniformly distributed within TiN matrix. TiN and Ti/TiH_2_ pairs serve as the light absorber, leading to a temperature of 283 °C under a light intensity of only 19 sun. Furthermore, the introduction of TiH_2_ clusters both thermodynamically and kinetically promotes the detachment of H atoms from MgH_2_, forming new Ti─H bonds inside the TiH_2_ matrix. Consequently, the homogeneous distribution of TiN_x_O_y_ and Ti/TiH_2_ within TiN matrix enables the uniform integration of photothermal and catalytic effect at a nanoscale, allowing localized heat to initiate catalysis and achieve a hydrogen capacity of 6.1 wt.% under a light intensity of 27 sun. Therefore, this work provides a rational design to realize solar‐driven hydrogen storage of metal hydrides by in situ constructing the photothermal‐catalytic coupling interface.

## Conflict of Interest

The authors declare no conflict of interest.

## Supporting information

Supporting Information

## Data Availability

The data that support the findings of this study are available in the supplementary material of this article.
