# Supplementary material for: In Situ Construction of Interface with Photothermal and Mutual Catalytic Effect for Efficient Solar‐Driven Reversible Hydrogen Storage of MgH2
Source: Adv Sci (Weinh). 2024 Mar 22;11(22):2400274. doi: 10.1002/advs.202400274 (PMC11165547; doi:10.1002/advs.202400274)
Supplement: Supplementary file 1 — Supporting Information [file ADVS-11-2400274-s001.pdf]

ADVANCED SCIENCE
Open Access

Supporting Information

for *Adv. Sci.*, DOI 10.1002/advs.202400274

In Situ Construction of Interface with Photothermal and Mutual Catalytic Effect for Efficient Solar-Driven Reversible Hydrogen Storage of MgH_2

Xuechun Hu, Xiaowei Chen, Xiaoyue Zhang, Yang Meng, Guanglin Xia, Xuebin Yu*, Dalin Sun and Fang Fang**

Supporting Information

In-situ interface construction with photothermal and mutual catalysis for efficient solar-driven reversible hydrogen storage of MgH_2

Xuechun Hu, Xiaowei Chen, Xiaoyue Zhang, Yang Meng, Guanglin Xia, Xuebin Yu*, Dalin Sun, Fang Fang**

X. C. Hu, Dr. X. Y. Zhang, Y. Meng, Prof. G. L. Xia, Prof. X. B. Yu, Prof. D. L. Sun, Prof. F. Fang
Department of Materials Science, Fudan University, Shanghai 200433, P. R. China
E-mail: xiaguanglin@fudan.edu.cn; yuxuebin@fudan.edu.cn; f_fang@fudan.edu.cn
Prof. F. Fang
Department of Materials Science, Fudan University, Shanghai 200433, Yiwu Research Institute of Fudan University, Yiwu City, Zhejiang 322000, P. R. China
Prof. X. W. Chen
Department of Physics, Jimei University, Xiamen 361021, P. R. China

Experimental Section

Synthesis of flower-liked TiO_2 microspheres

In a typical synthesis, 4 mL tetrabutyl titanate (TBOT, Aladdin, ≥ 99.0%) was added into 60 mL of acetic acid (GR, ≥ 99.8%) with mild stirring, followed by ultrasonic for several minutes at room temperature to accelerate the complexation reaction. The obtained light-yellow gel-like mixture was then transferred to a 100 mL stainless steel autoclave and treated at 160 °C for 3 h. The collected products were centrifugally separated and further washed several times by ethanol, and then vacuum-dried overnight at 80 °C to yield white powder. The resultant powder

samples were pre-grinded and calcined at 500 °C for 3 h to obtain the final products as flower-liked TiO_2 microspheres.

Synthesis of carbon coated TiN@N-TiO_2-*x* (TNT*x*)

The as-synthesized TiO_2 was mixed with melamine ($C_3H_6N_6$, CP, ≥ 99.0%) at a mass ratio of 1:*x* by facile grinding and then calcined at 750 °C for 3 h with a heating rate of 3 °C min^{-1} at a flow of 5% H_2/N_2. The thus-obtained dark black products were denoted as TiN@N-TiO_2-*x* (TNT*x*, *x* = 3, 5, 20).

Preparation of MgH_2 catalyzed by TiO_2, TNT*x*, and TiN

The TiN@N-TiO_2-*x* catalyst with a mass ratio of 10 wt.% was mixed with MgH_2 (Alfa Aesar, 98%) by mechanically milling for 12 h under a H_2 pressure of 50 atm. The ball-to-powder ratio was controlled to be 100:1 and the milling speed was kept at 500 rpm during the whole process. The resulting composite was denoted as MgH_2-TNT*x*. For comparison, as-synthesized TiO_2 and commercial TiN (Aladdin, ≥ 99.90%) were also mixed with MgH_2 under identical conditions. All the operations were carried out in an Ar-filled glovebox with the water and oxygen contents below 0.01 ppm.

Characterizations

The phase composition was determined by X-ray diffraction (XRD, D8 Advance, Bruker AXS) with Cu Kα radiation (λ = 1.542 Å). The samples were protected by amorphous tapes (with a broad peak around 2θ ≈ 20°) in an Ar-filled glovebox to avoid oxidation. The morphologies and element distribution were characterized by scanning electron microscopy (SEM, JEOL 7500FA, Japan) and transmission electron microscopy (TEM, JEOL JEM-2100F, Japan) equipped with an energy-dispersive X-ray spectroscopy (EDS) analysis unit. The X-ray photoelectron spectroscopy (XPS) was carried out on a Thermo Scientific K-Alpha+ system

equipped with dual X-ray sources, adopting an Al Kα anode with a hemispherical energy analyzer. The UV-*vis*-NIR Spectrophotometer (Lambda 35, Perkin-Elmer, USA) was taken to determine the absorption spectra of samples in the wavelength range of 200~2000 nm. A 300 W Xe lamp (CEL-HXF300-T3, Beijing China Education Au-light Co., Ltd.) was used as the light source, and an optical power meter (PL-MW2000, Perfect Light Co., Ltd.) was adopted to measure the light intensity. A short-wave infrared thermometer (DGE44N, range: 75~650 °C, DIAS, Germany) was used to measure the surface temperature of the composites under light irradiation with a recording time step of 1 second. The light reactor cell (manufactured by Beijing Century Senlong experimental apparatus Co., Ltd.) was equipped with a Sapphire window with a light transmittance of ~90-95% on the top surface and several fiber glass filter on the ground to achieve effective thermal management.

Hydrogen Storage Measurements

Hydrogen storage performance of the samples was evaluated by a home-built high-pressure gas sorption apparatus (HPSA-auto), which was carefully calibrated by adopting $LaNi_5$ as a reference sample in terms of hydrogen storage capacity and guaranteed an accuracy of ±1%. The hydrogen gravimetric capacity was calculated on the total mass of test sample.

In the term of electric heating test, approximately 25 mg of test samples were heated from room temperature to 450 °C at different heating rate under an initial pressure below 0.0001 bar in the non-isothermal desorption process. The isothermal H_2 desorption and absorption test was handled by rapidly heating up to the target temperature followed by keeping at the preset temperature. In the isothermal H_2 absorption experiment, approximately 80-90 mg of samples were used for hydrogenation under the hydrogen pressure of 50 bar.

In the term of light irradiation test, 25 mg of samples were pressed into pieces with a diameter of 15 mm under a pressure of 5 tons to avoid powder spattering and ensure the stability of the test and the comparability of the experimental results. The top of the light reactor is a sapphire

window with a light transmittance of more than 95%, and the light reactor was connected to the HPSA-auto to record real-time hydrogen capacity. The light intensity was adjusted by tuning the current of the light source with a fixed distance between the light source and the reactor, which could avoid the measure error caused by the different irradiation area due to the different distance. To reduce the heat loss, a layer of SiO_2 glass fiber with low thermal conductivity was added to the bottom of the device and a tailor-made SiO_2 glass fiber ring with an inner diameter of 16mm was introduced to restrict the pellet's movement under light irradiation in the vacuum, thereby preventing heat dissipation through the sidewall. The cycling test was carried out at a light intensity of 35 sun with an initial desorption pressure below 0.0001 bar and a H_2 pressure of 50 bar for adsorption.

FDTD Simulation

To clarify the mechanism of plasmon-enhanced photothermal properties, the electric field distributions of the TiN@N-TiO_2 and TiO_2 were simulated by the finite-difference time-domain (FDTD) simulation performed on FDTD solutions 8.6 (Lumerical Solutions Inc., Vancouver, Canada). The profiles of electric fields (described by $|E|^2/|E_0|^2$) were obtained when the incident light was perpendicular to one of the facets[1]. The simulation structure of TiN@N-TiO_2 was composed of N-TiO_2 core (diameter: 40 nm), periodical TiN nanoparticles on the surface (diameter: 10 nm) and the top layer covered with amorphous carbon coating (thickness: 2 nm). The dielectric constant of TiN, and C was obtained from the FDTD database taken from the Palik model[2]. The permittivity of N-TiO_2 was obtained from previous literatures, and the dielectric functions with the minimum root-mean-square error (RMSE) of experimental results was calculated with Eq. (S1) as follow[3] using the model parameters of ellipsoid fitting:

$$\varepsilon_{N-TiO_2} = \varepsilon_\infty - \frac{E_p^2}{E^2+i\Gamma_D E} + \frac{f_{LSPR}E_{LSPR}^2}{E_{LSPR}^2-E^2-i\Gamma_{LSPR}E} + \frac{f_{int}E_{int}^2}{E_{int}^2-E^2-i\Gamma_{int}E} \quad \text{(Equation S1)}$$

where ε_∞ is the high frequency dielectric constant; E_p (the Drude plasma energy of free electron) =0.94 eV; f_{LSPR}(strength of the LSPR oscillator) =3.75; E_{LSPR}(resonance energy of the LSPR oscillator) =1.37eV, f_{int} (strength of the interband transitions) =3.73; E_{int}(resonance energy of the interband transitions) =4.74 eV.

The simulation regions (x, y, z) = (-50:50, -50:50, 0) nm was divided into uniform Yee cells with perfectly matched layer boundary (PML) in x-, y-, and z-directions. An incident plane wave was propagated from the top along z axis and polarized along the x axis, and a wavelength of 540 and 1200 nm light was used for calculation. The process was performed at 1 nm mesh resolution and 500 fs time. A 2D electric field monitor located at y = 0 was used to investigate the electric field distribution across the 3D core-shell structure.

Theoretical calculations

The density functional theory (DFT) calculations were performed using the VASP code[4]. The electron-ion interaction was modeled using the projector-augmented wave (PAW) method[5]. The exchange-correlation functional was described using the Perdew-Burke-Ernzerhof (PBE) generalized gradient approximation (GGA) [6]. A plane-wave energy cutoff of 400 eV was employed for all calculations. To minimize interactions between neighboring layers, a large vacuum slab (~26 Å) was used along the z-direction. A Gaussian smearing technique with a small SIGMA value of 0.05 was applied in all the calculations. The Wulff-like models for TiH_2 and MgH_2 nanoparticles were generated using the bulk cut nanoparticle model (BCN-M) computational tool [7]. Geometric optimization was performed by relaxing all atoms until the residual force was less than 0.02 eV/Å. Ab initio molecular dynamics (AIMD) simulations were carried out using the Nose-Hoover method to solve the ionic motion equations, with a time step of 1.0 fs. The system was equilibrated at 1200 K for 60 ps in the canonical ensemble (NVT). The climbing image nudged elastic bond (CI-NEB) method[8] was utilized to investigate the pathways and energy barriers associated with hydrogen release in the given system.

Supplementary Figures

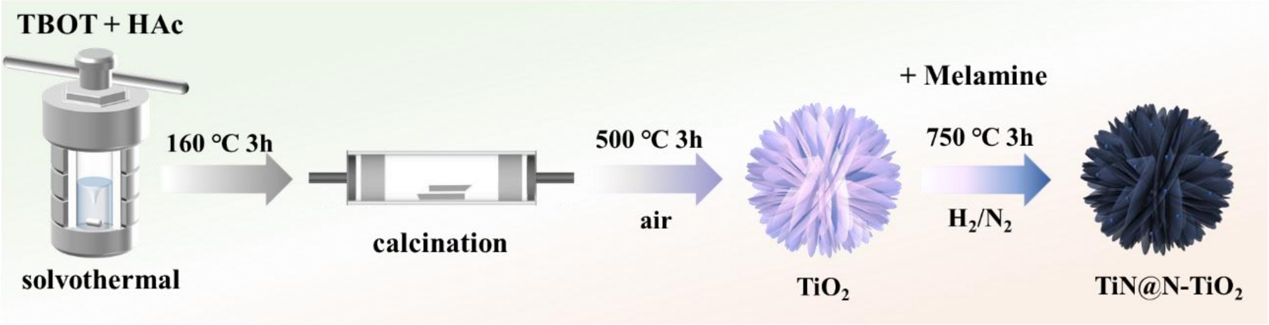

Figure S1. Schematic illustration of the preparation process of TiN@N-TiO_2

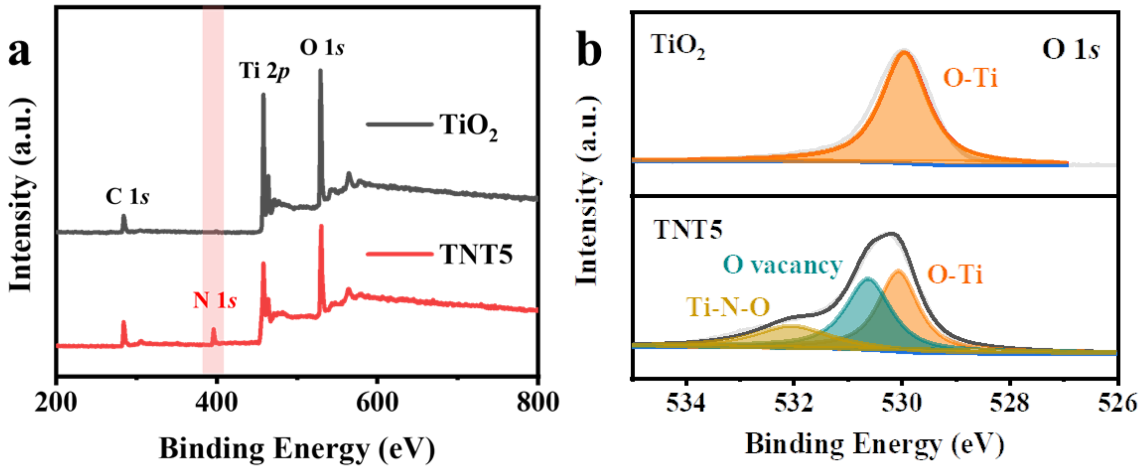

Figure S2. (a) XPS survey spectra and (b) high-resolution O 1*s* XPS spectrum of the as-synthesized TiO_2 and TNT5.

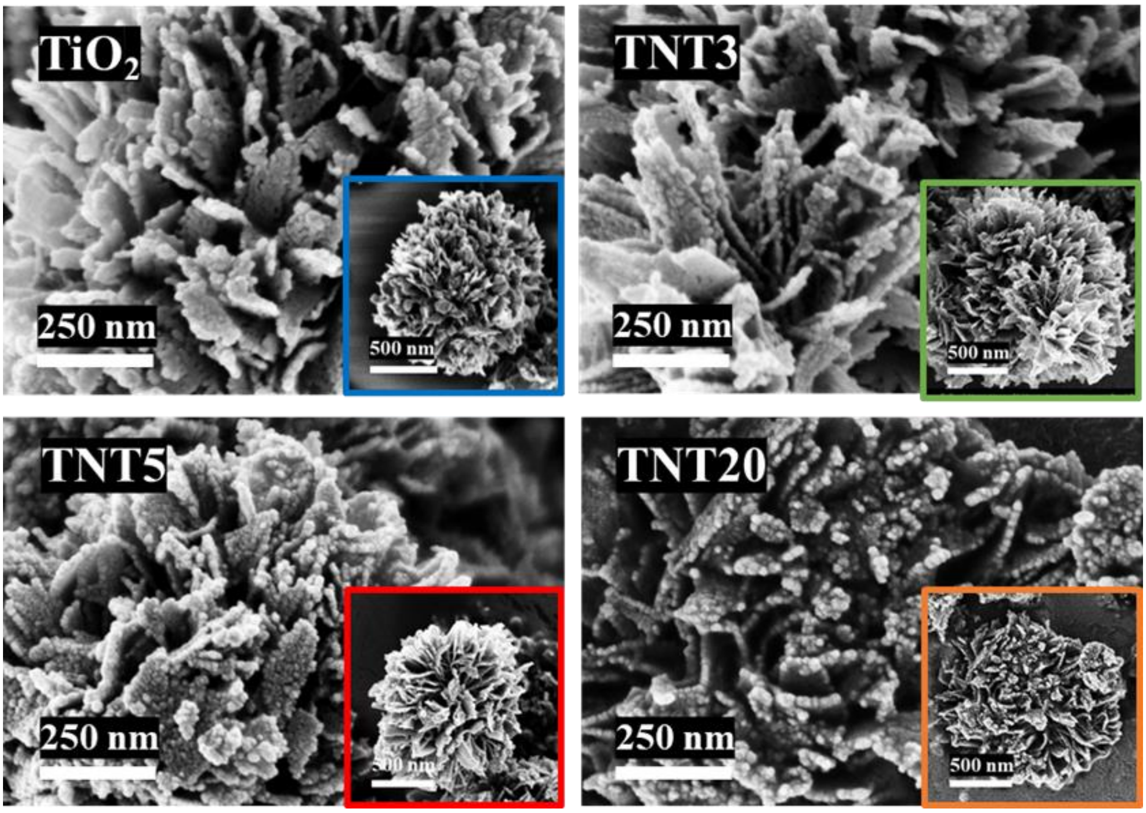

Figure S3. SEM images of the as-synthesized TiO_2, TNT3, TNT5, and TNT20.

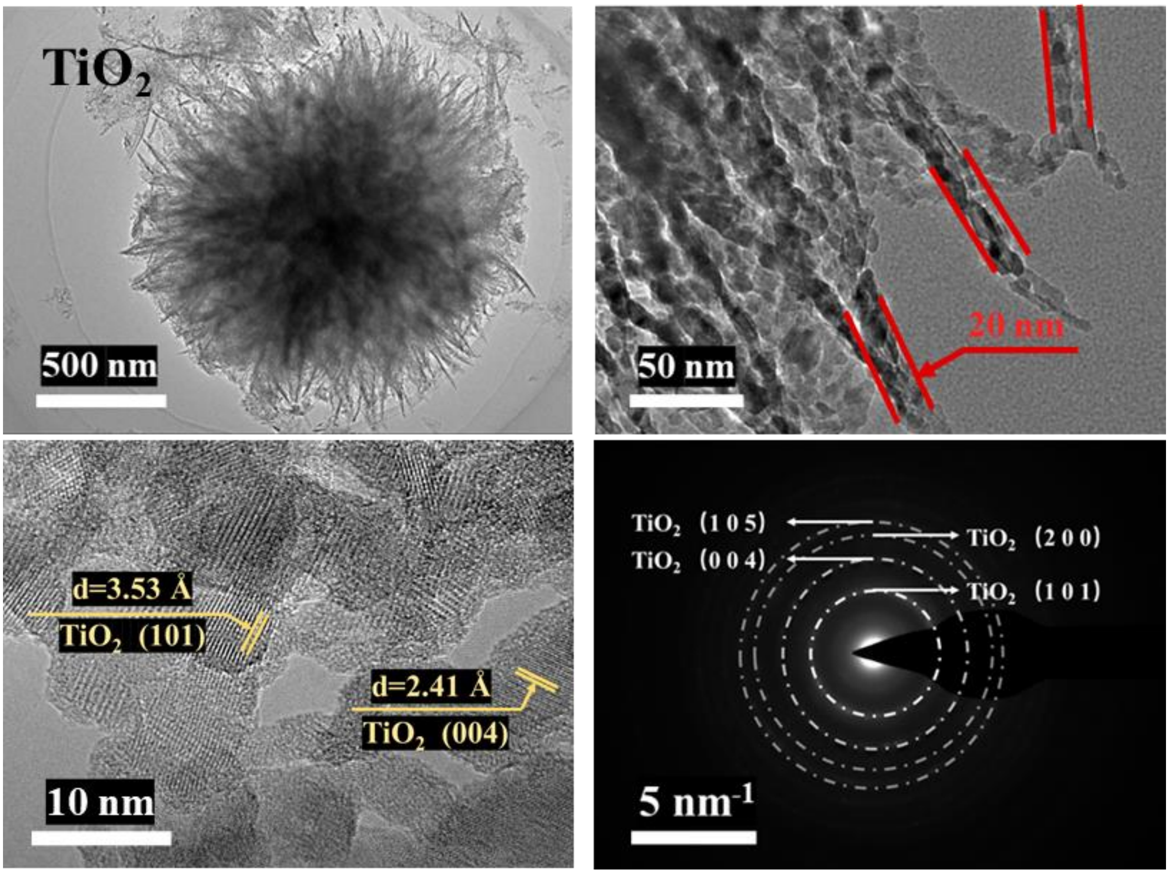

Figure S4. HRTEM images and SAED patterns of the as-synthesized TiO_2.

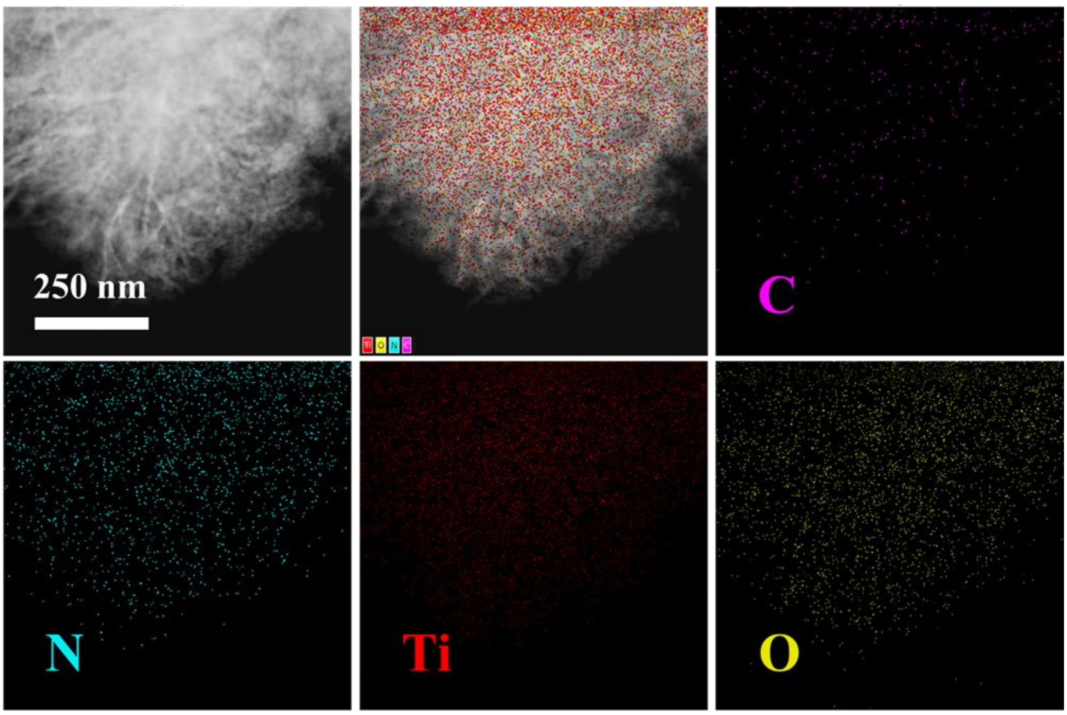

Figure S5. Elements mapping images of Ti, N, O and C in TNT5.

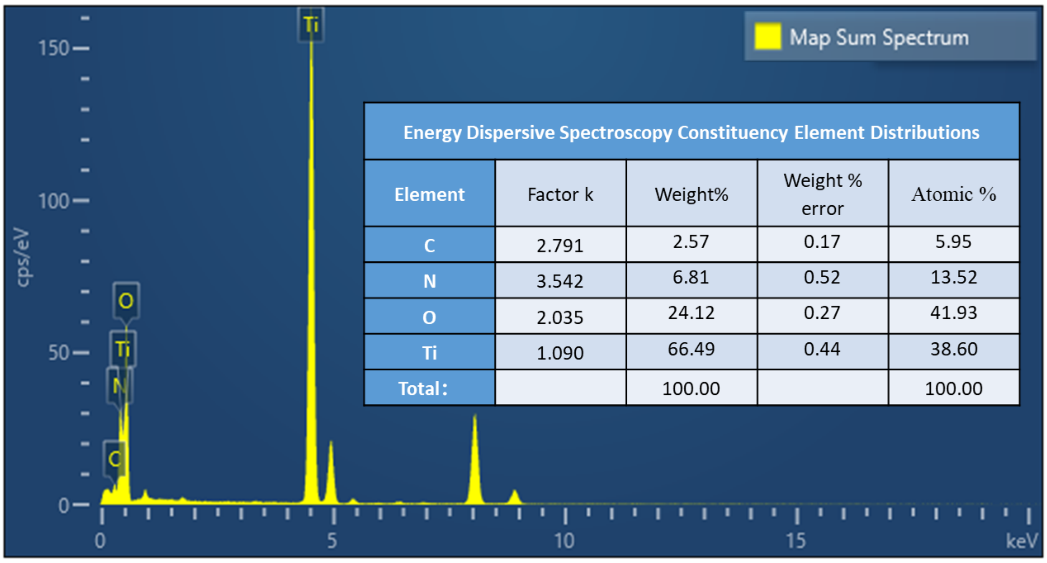

Energy Dispersive Spectroscopy Constituency Element Distributions

Element	Factor k	Weight%	Weight % error	Atomic %
C	2.791	2.57	0.17	5.95
N	3.542	6.81	0.52	13.52
O	2.035	24.12	0.27	41.93
Ti	1.090	66.49	0.44	38.60
Total:		100.00		100.00

Figure S6. The relative elemental content analysis of TNT5.

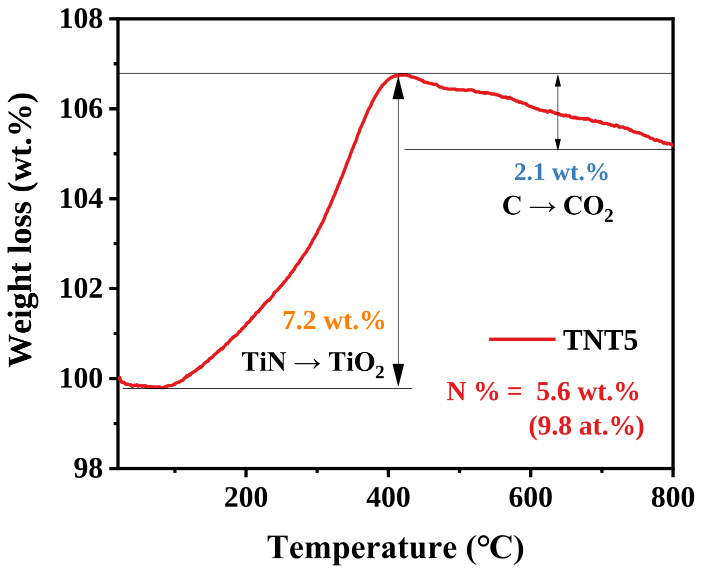

Figure S7. Measured TG curves as a function of temperature for TNT5.

Table S1. N atom content of TiN@N-TiO_2-*x* samples..

**Sample**	**TNT3**	**TNT5**	**TNT20**
**N (at.%)**	7.91	10.16	13.52
**Error (%)**	0.23	0.27	0.43

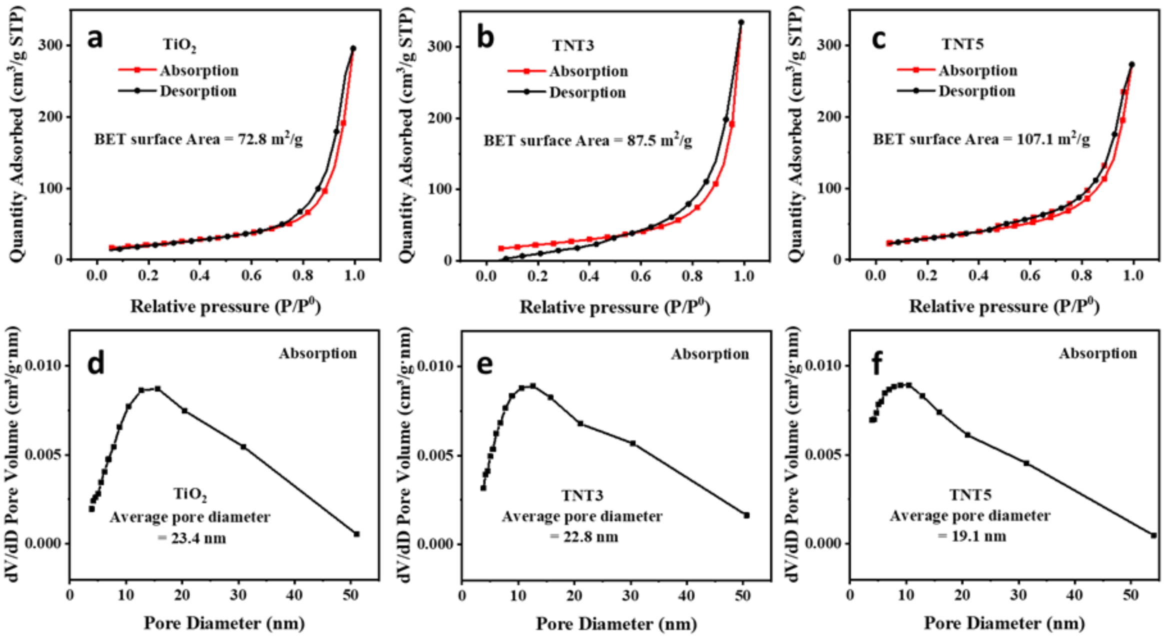

Figure S8. (a) N_2 adsorption-desorption isotherms and (b) the relative pores size distribution of as-synthesized TiO_2, TNT3, TNT5, and TNT20.

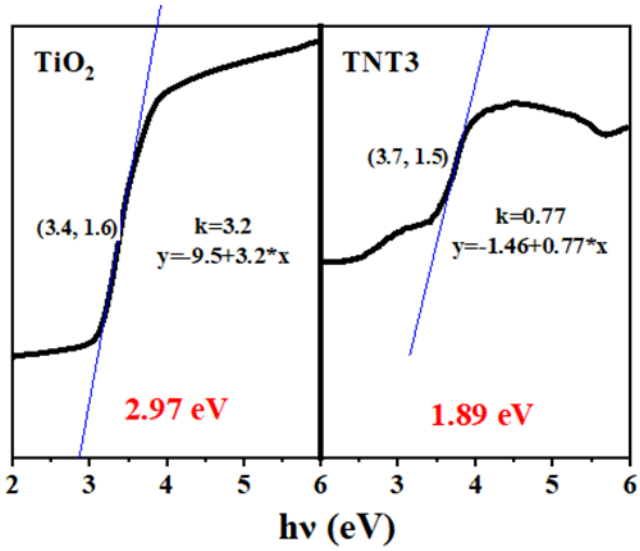

Figure S9. Analysis of absorption spectra of the as-synthesized TiO_2 and TNT3 for the indirect electronic transition $(\alpha h\nu)^{1/2}$ as a function of the photon energy (eV). Spectral analysis is conducted using a Tauc plot and the base lines are used to determine Eg [9].

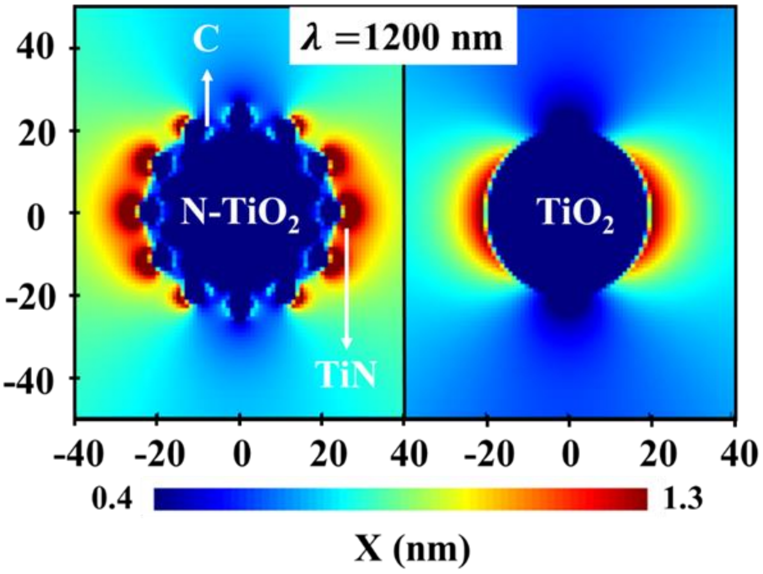

Figure S10. Theoretical FDTD simulated localized electric field enhancement profiles obtained under the wavelength of 1200 nm light, corresponding to the NIR region.

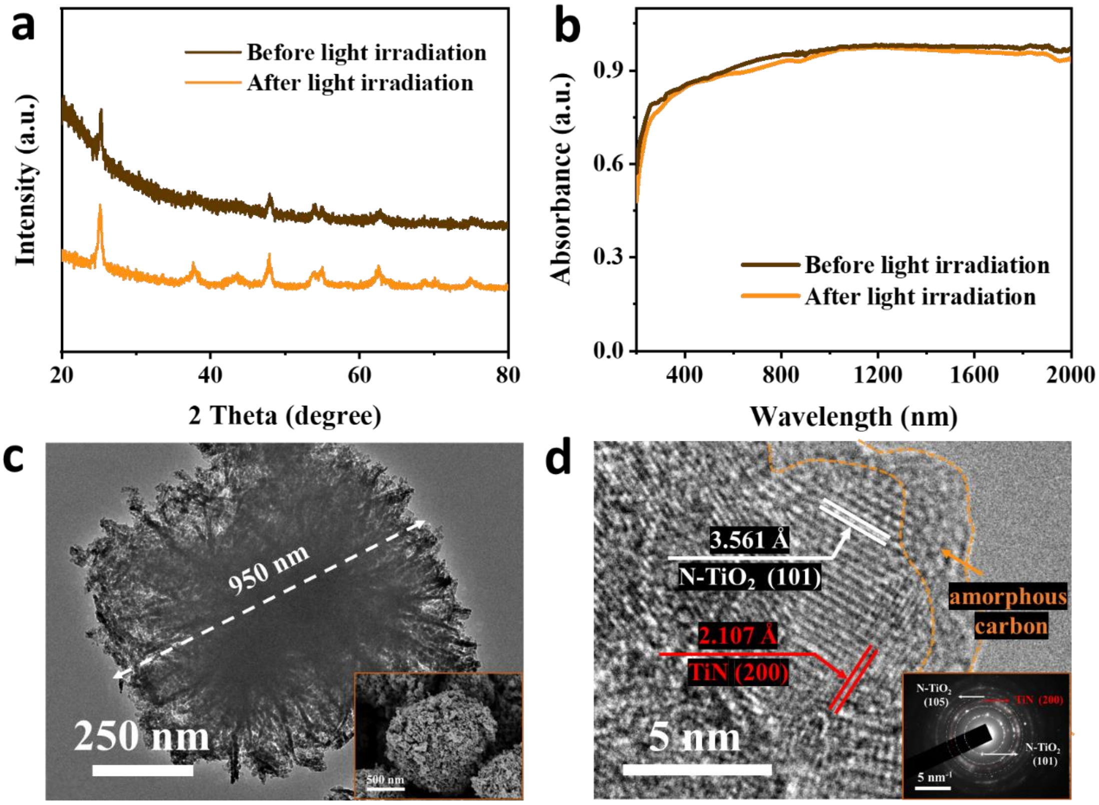

Figure S11. (a) XRD patterns, (b) UV-vis-NIR absorption spectra, (c) TEM, (d) HRTEM images and corresponding SAED images of TiN@N-TiO_2-*x* before and after light irradiation

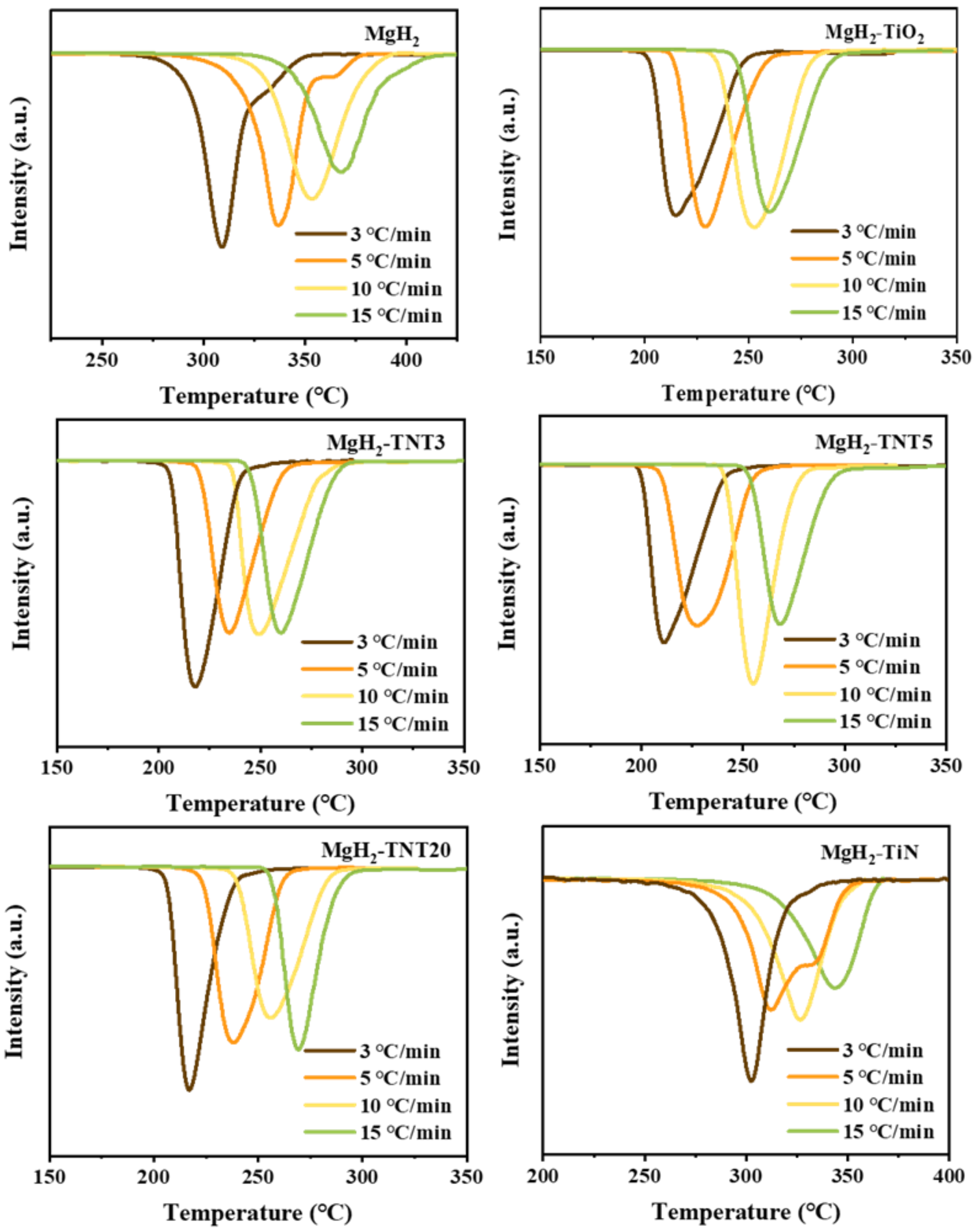

Figure S12. The derivative curves corresponding to the TPD curves of MgH_2 catalyzed by as-synthesized TiO_2, TNT*x*, and TiN, including ball-milled MgH_2 for comparison.

Table S2. The apparent activation energy (E_a) of MgH_2 catalyzed by TiO_2 ,TNT*x*, and TiN calculated by the Kissinger's equation[10].

**Materials**	**MgH_2**	**+TiO_2**	**+TNT3**	**+TNT5**	**+TNT20**	**+TiN**

E_a（kJ/mol）	136.8	80.6	75.2	74.3	85.8	107.8
R^2	0.9928	0.9954	0.9933	0.9975	0.9940	0.9929

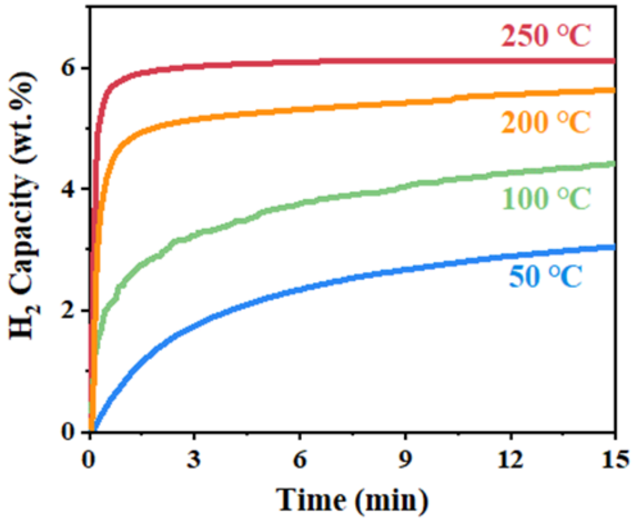

Figure S13. Isothermal H_2 adsorption curves of MgH_2 catalyzed by TNT5 at various temperatures.

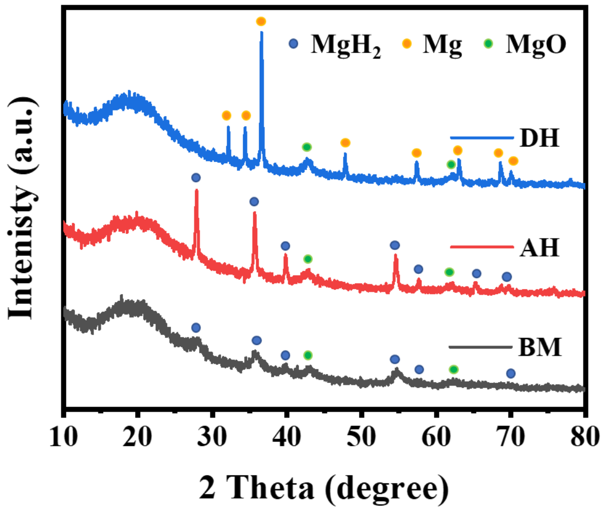

Figure S14. XRD patterns of the ball-milled (BM), dehydrogenated (DH), and re-hydrogenated (AH) MgH_2 catalyzed by TNT5.

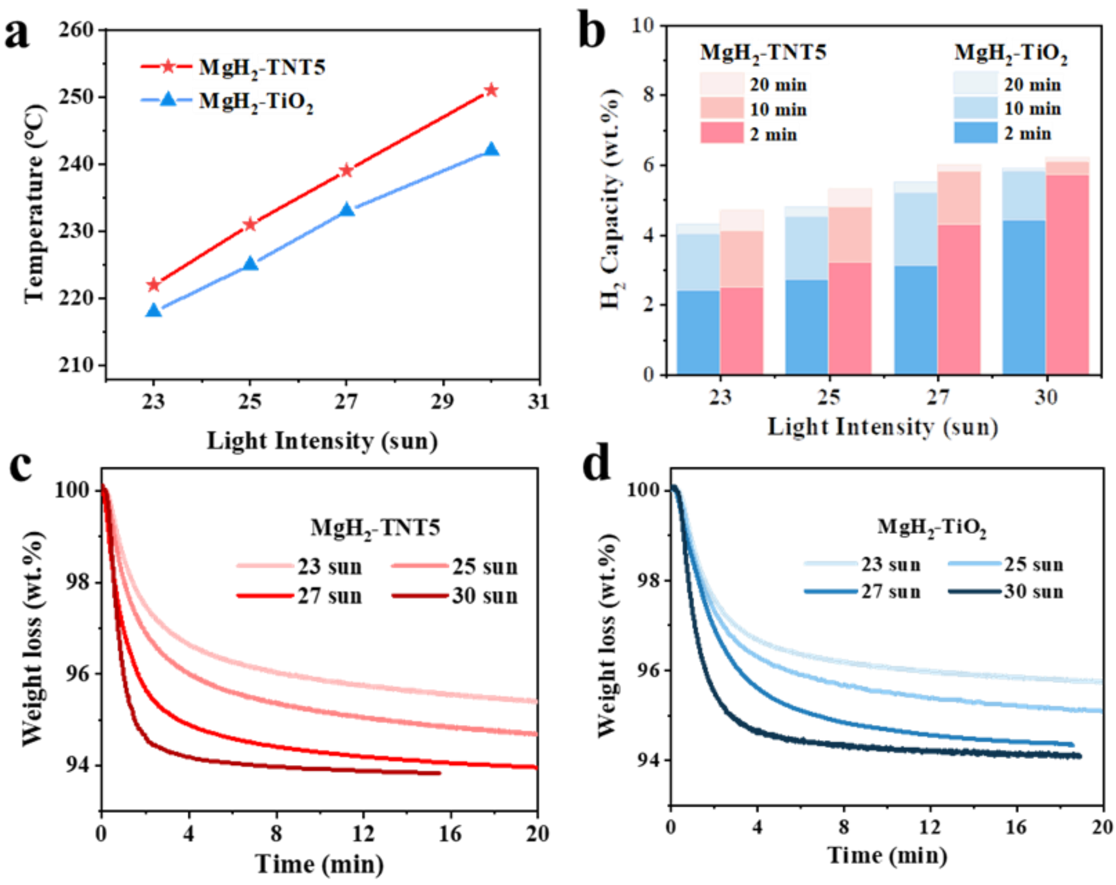

Figure S15. (a) The stable temperatures at 20 min and (b) the corresponding H_2 desorption curves of MgH_2 under the catalysis of TNT5 and TiO_2 under various light intensities. (c, d) Isothermal H_2 desorption curves of MgH_2 catalyzed by TNT5 and TiO_2 at various light intensity irradiation.

Table S3. Comparison for solar-driven reversible H_2 desorption of MgH_2 catalyzed by various catalysts.

Catalyst	Au	Cu@MXene	TiN@N-TiO_2
Photothermal temperature (catalyst)	None	260 °C (14 sun)<br>325 °C (19 sun)	245 °C (16 sun)<br>320 °C (22 sun)
Photothermal temperature (+MgH_2)	100 °C (1 sun)	208 °C (26 sun)<br>225 °C (31 sun)	231 °C (25 sun)<br>250 °C (30 sun)
H_2 desorption performance	Negligible	**26 sun**<br>60 min-3.9 wt.%<br>**31 sun**<br>10 min-5.1 wt.%;	**25 sun**<br>10 min-4.5 wt.%<br>**30 sun**<br>10 min-6.2 wt.%
Reversible capacity	None	**40 sun**	**35 sun**

		30th -5.9 wt.%	15th -6.05 wt.%
Capacity retention	None	30th -91%	15th -95%

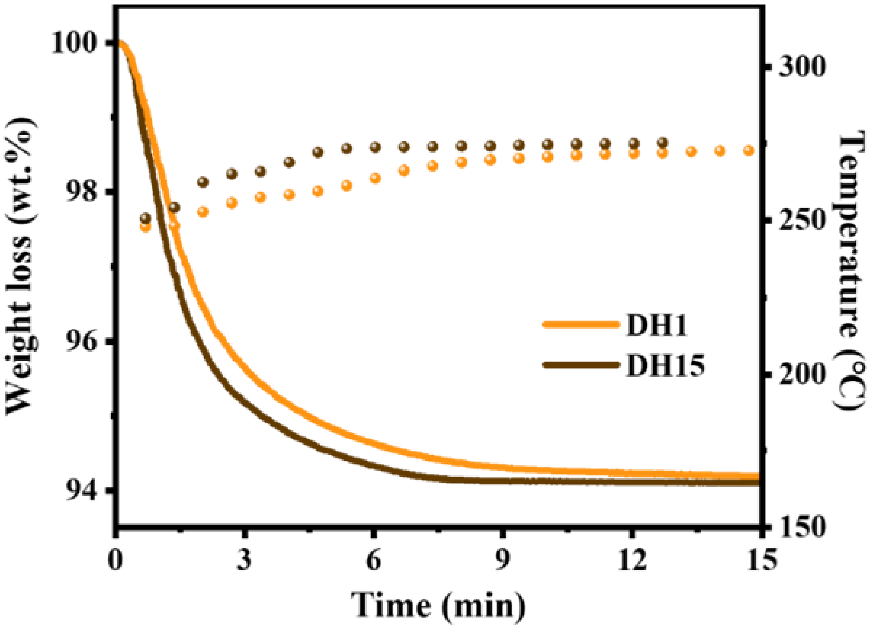

Figure S16. Comparison of surface temperature and dehydrogenation kinetics of MgH_2 catalyzed by TNT5 under solar irradiation between the 1st and 15th cycles.

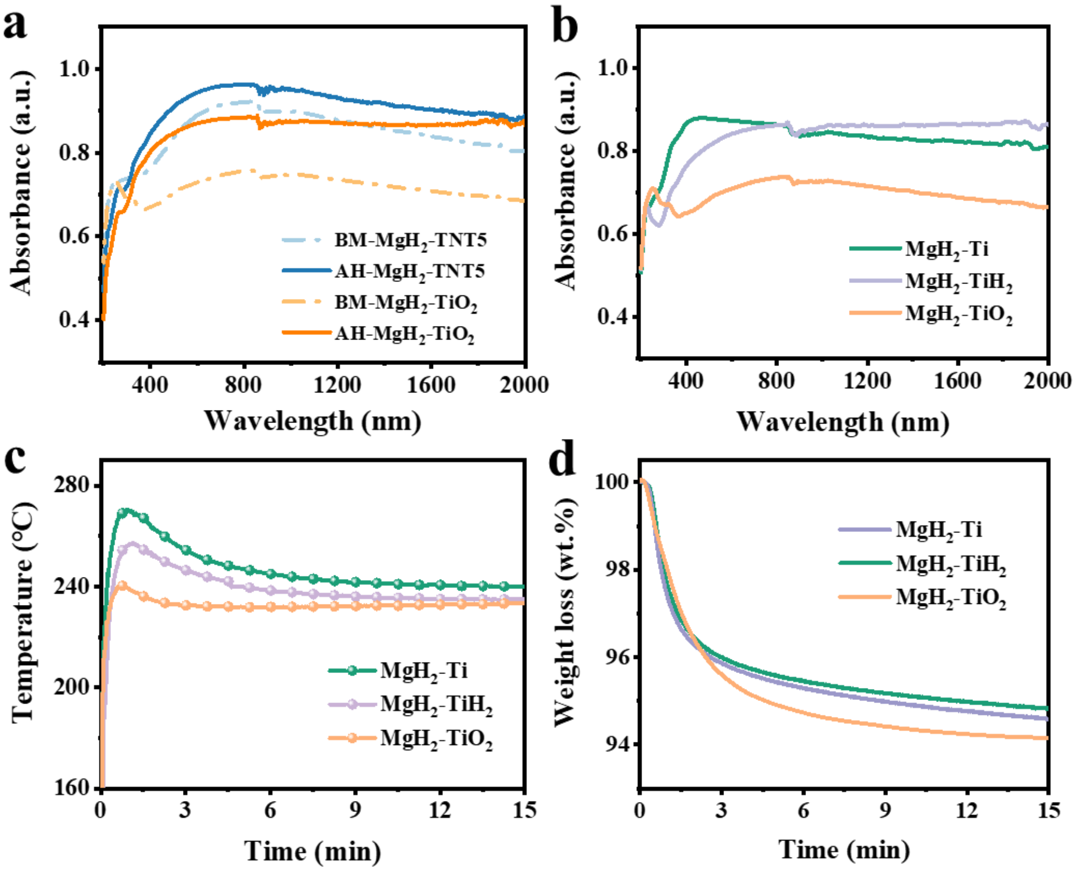

Figure S17. (a) UV–*vis*–NIR absorption spectra of MgH_2 catalyzed by TiO_2 and TNT5 after cycling compared with ball-milled state. (b) UV–*vis*–NIR absorption spectra of MgH_2 catalyzed by Ti, TiH_2 and TiO_2. (c, d) The surface temperature curves and solar-driven H_2 desorption curves of MgH_2 catalyzed by Ti, TiH_2 and TiO_2 at the light intensity of 27.5 sun.

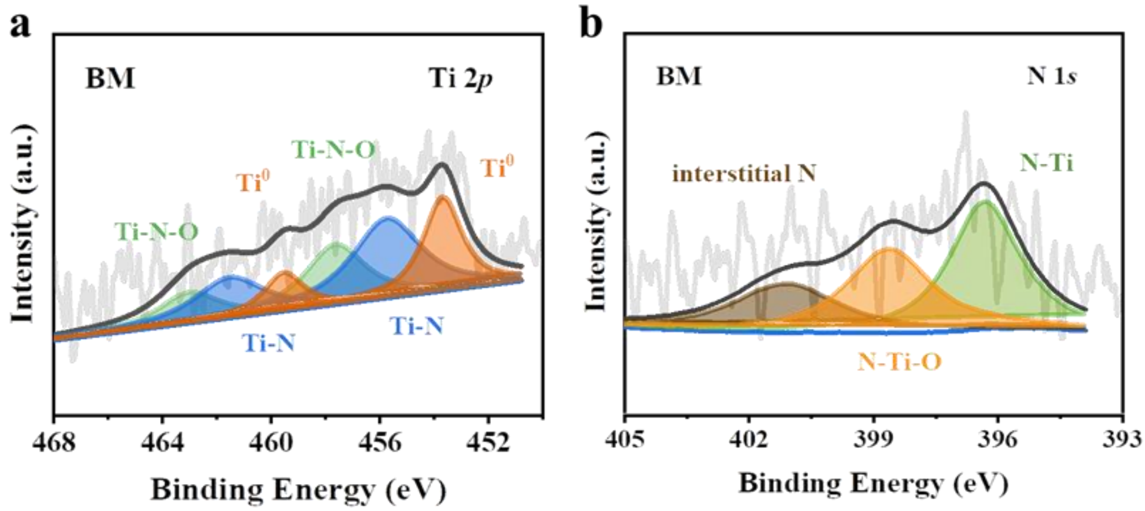

Figure S18. High-resolution (a) Ti 2*p* and (b) N 1*s* XPS spectra of MgH_2 catalyzed by TNT5 after ball-milling.

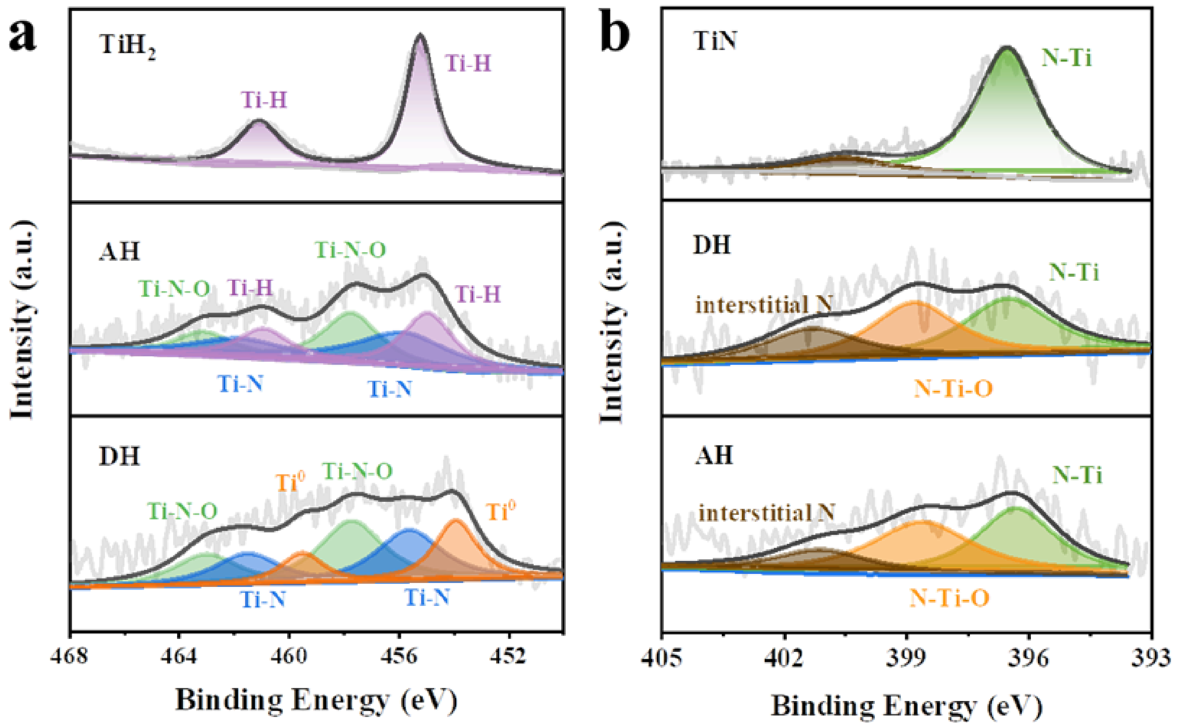

Figure S19. High-resolution Ti 2*p* and N 1*s* spectra of MgH_2 catalyzed by TNT5 at various states, including dehydrogenated (DH), hydrogenated (AH), including commercial TiH_2 and TiN for comparison.

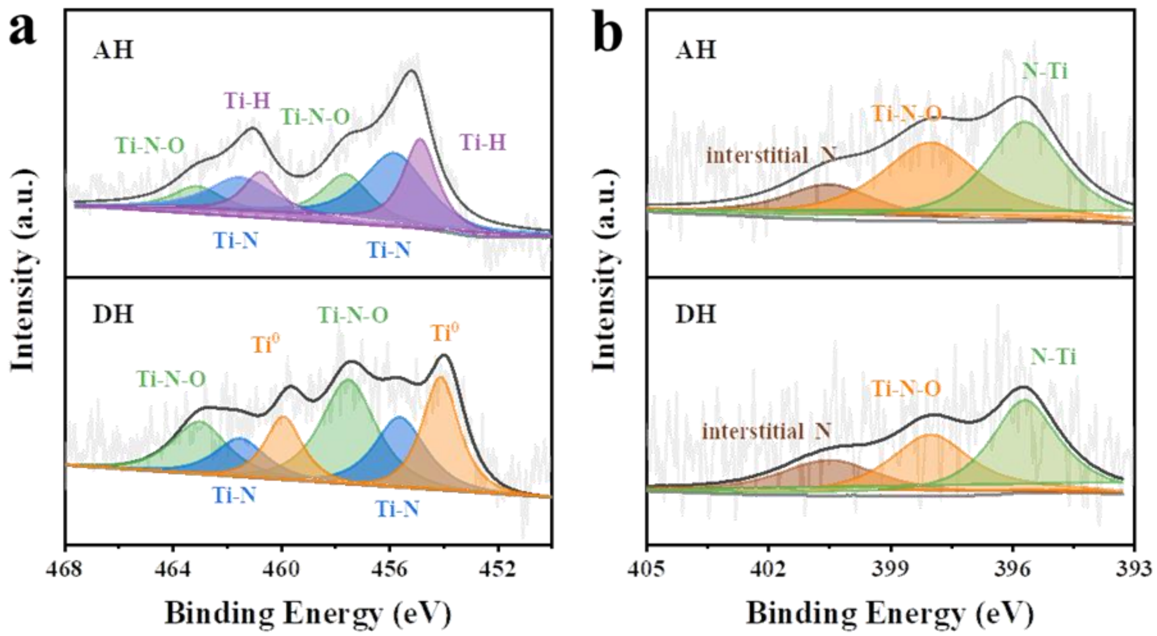

Figure S20.High-resolution (a) Ti 2*p* and (b) N 1*s* XPS spectra of MgH_2 catalyzed by TNT5 after cycling under light irradiation.

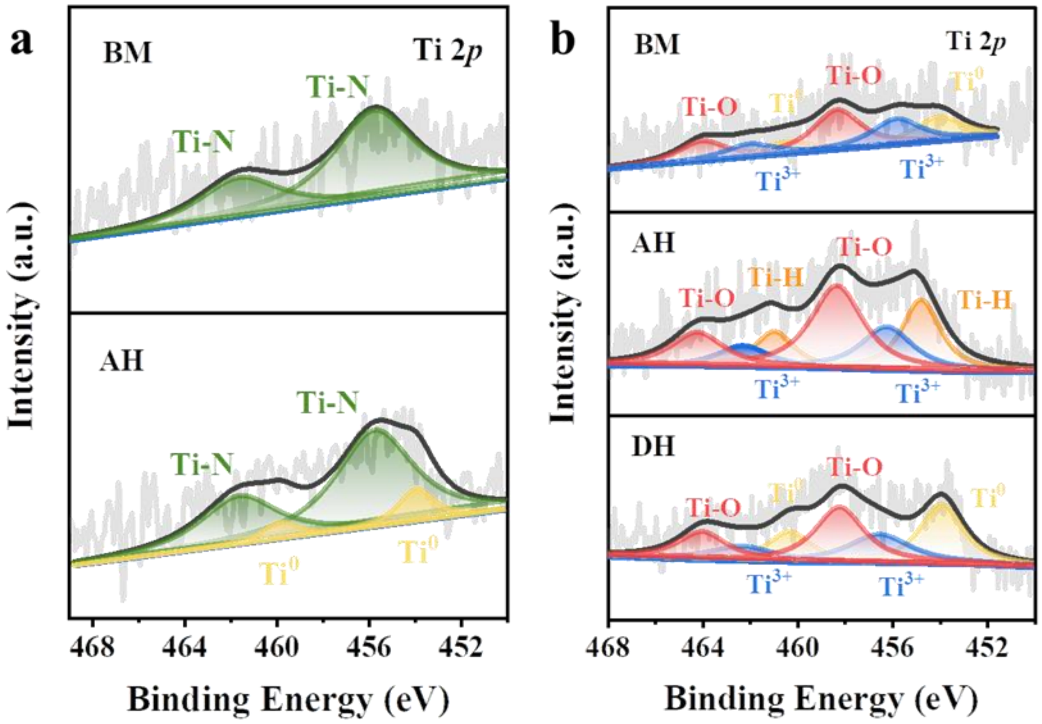

Figure S21. High-resolution Ti 2*p* XPS spectra of MgH_2 catalyzed by (a) TiN and (b) TiO_2 at various states, including ball-milled (BM), dehydrogenated (DH) and re-hydrogenated (AH) states.

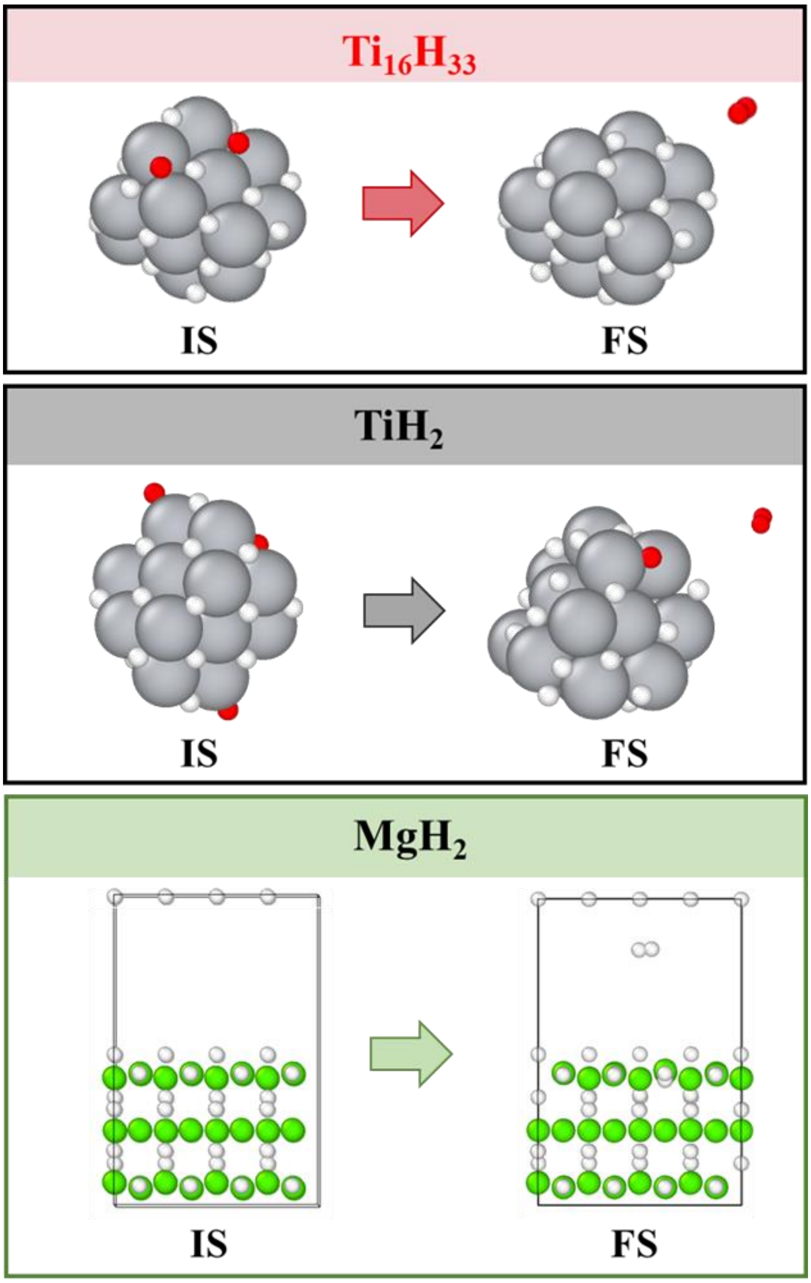

Figure S22. Schematic illustration of dehydrogenation pathway of $Ti_{16}H_{33}$, TiH_2 and MgH_2.

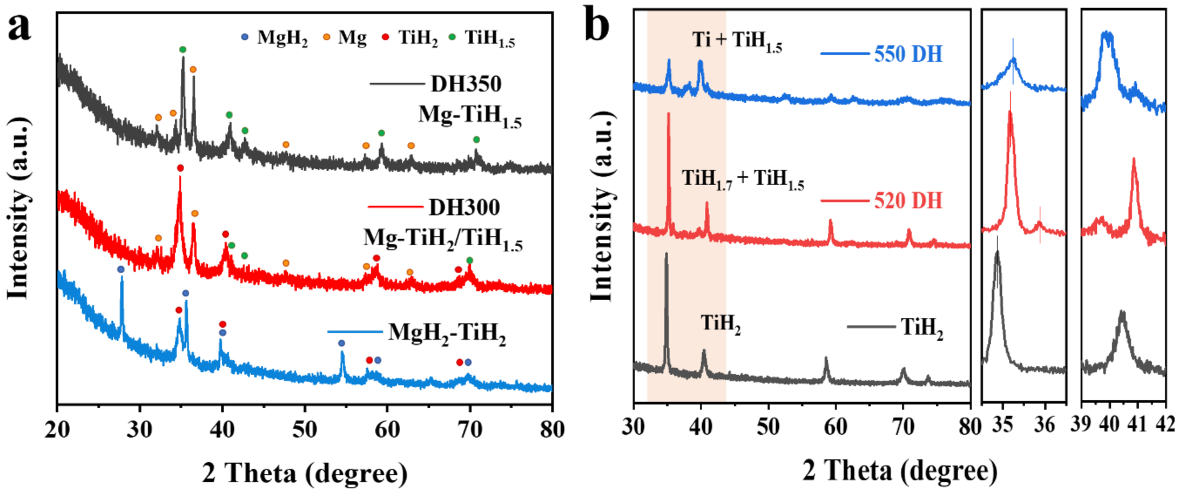

Figure S23. XRD patterns of dehydrogenation products of (a) MgH_2-TiH_2 and (b) pristine TiH_2 at different temperatures.

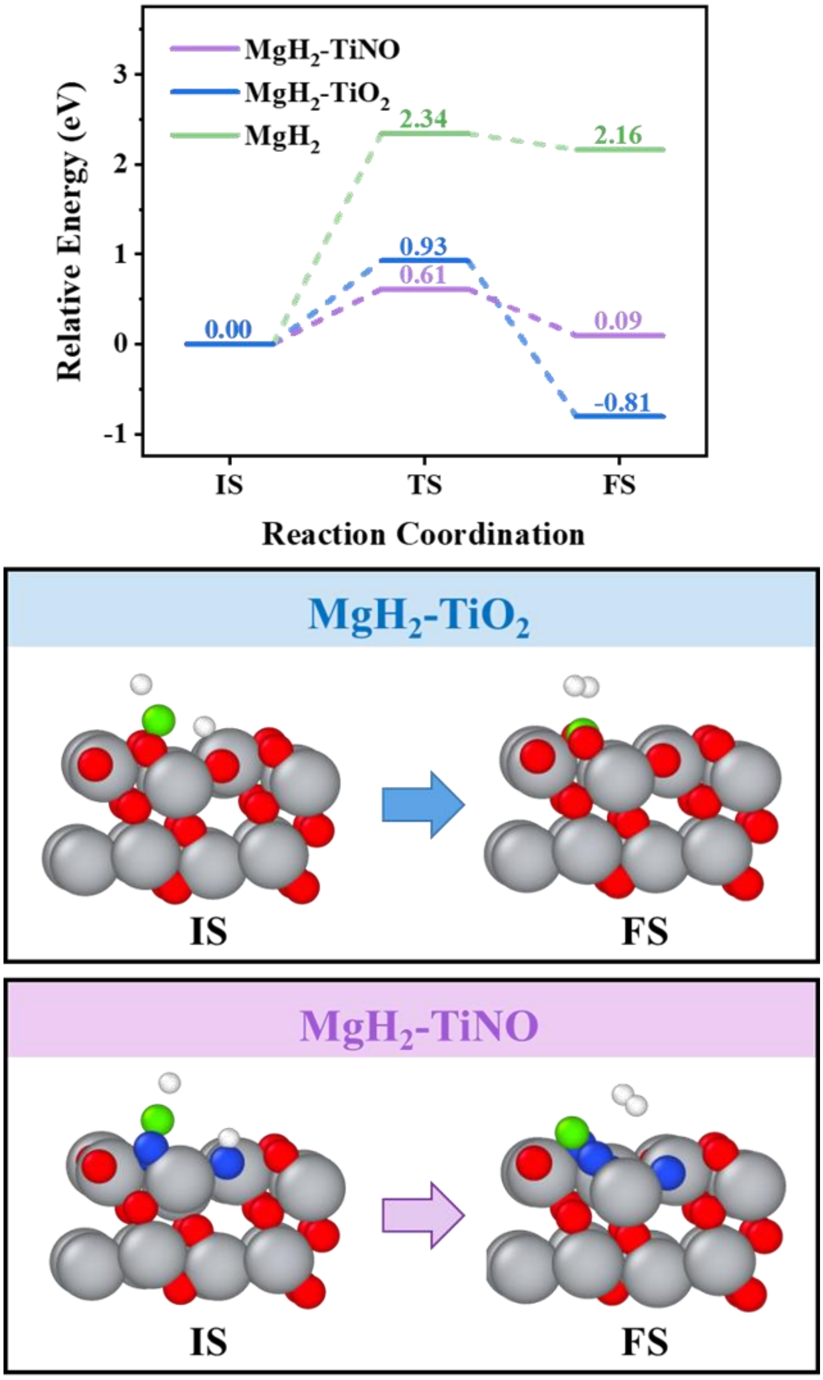

Figure S24. Energy profile and Schematic illustration of H_2 desorption pathway of MgH_2 under the catalysis of TiO_2 and TiNO [11].

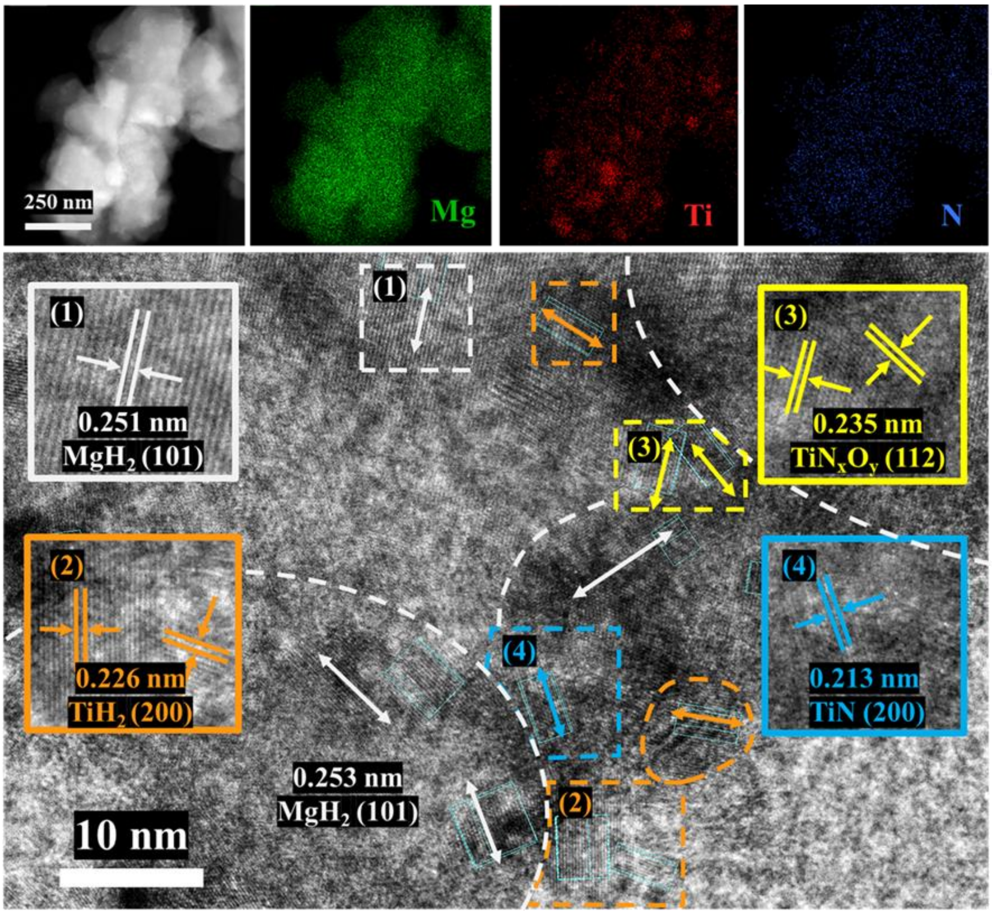

Figure S25. HRTEM and elements mapping images of Mg, Ti, and N in MgH_2 catalyzed by TNT5 after cycling.

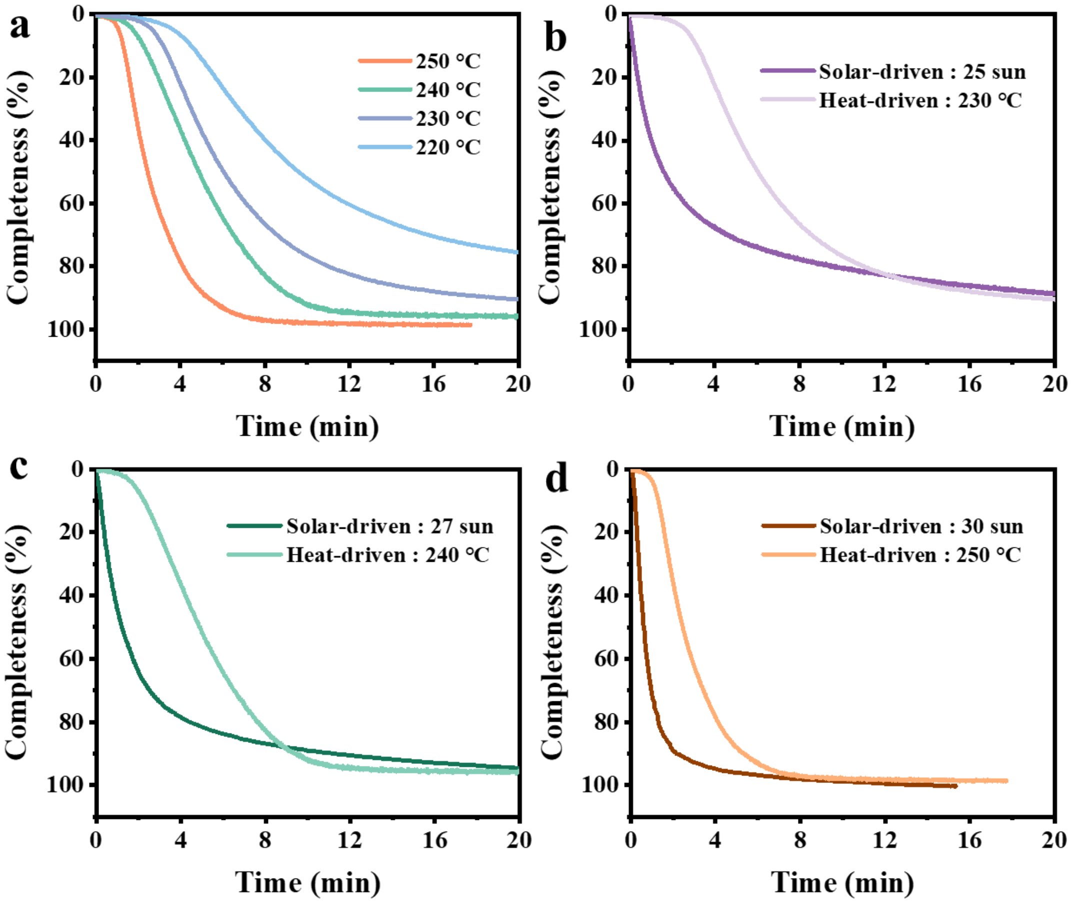

Figure S26. (a) Isothermal dehydrogenation curves at various temperature of MgH_2 catalyzed by TNT5. (b-d) The comparison of H_2 desorption curves of MgH_2 catalyzed by TNT5 using solar energy (light intensity of 25, 27 and 30 sun) and corresponding thermal heating (230, 240, 250 °C), respectively.

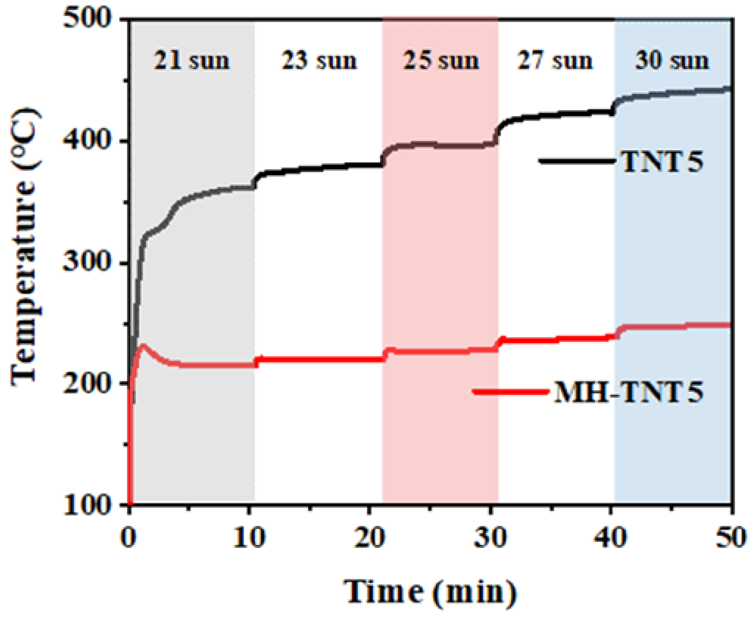

Figure S27. Comparison of the temperature curves of TiN nanoparticles and MgH_2 catalyzed by TNT5 over time under the various light irradiation conditions.

References

[1] L. Cheng, G. Zhu, G. Liu, L. Zhu, *Materials Research Express* **2020**, 7.

[2] M. N. Gadalla, K. Chaudhary, C. M. Zgrabik, F. Capasso, E. L. Hu, *Optics Express* **2020**, 28.

[3] J. Zhang, T. P. Chen, Y. C. Liu, Z. Liu, H. Y. Yang, *Solar Energy* **2017**, 142, 33.

[4] G. Kresse, J. Furthmüller, *Physical Review B* **1996**, 54, 11169.

[5] P. E. Blöchl, *Physical Review B* **1994**, 50, 17953.

[6] a)J. P. Perdew, K. Burke, Y. Wang, *Physical Review B* **1996**, 54, 16533; b)J. P. Perdew, K. Burke, M. Ernzerhof, *Physical Review Letters* **1996**, 77, 3865.

[7] D. González, B. Camino, J. Heras-Domingo, A. Rimola, L. Rodríguez-Santiago, X. Solans-Monfort, M. Sodupe, *The Journal of Physical Chemistry C* **2020**, 124, 1227.

[8] G. Henkelman, B. P. Uberuaga, H. Jónsson, *The Journal of Chemical Physics* **2000**, 113, 9901.

[9] J. S. Lee, K. H. You, C. B. Park, *Adv Mater* **2012**, 24, 1084.

[10] Y. Li, Y. Cheng, Y. Ye, R. Shen, *Journal of Thermal Analysis and Calorimetry* **2009**, 102, 605.

[11] F. Schweppe, M. Martin, E. Fromm, *Journal of Alloys and Compounds* **1997**, 253-254, 511.

[12] a)J. Graciani, S. Hamad, J. F. Sanz, *Physical Review B* **2009**, 80; b)B. Ni, G. Zhang, H. Wang, Y. Min, K. Jiang, H. Li, *Angewandte Chemie International Edition* **2022**, 62.
